# Serpina3c deficiency promotes obesity-related hypertriglyceridemia and inflammation through activation of the Hif1α*-*glycolysis axis in adipose tissue

**DOI:** 10.1042/CS20242610

**Published:** 2025-08-28

**Authors:** Jiaqi Guo, Zhenjun Ji, Yu Jiang, Ya Wu, Shaofan Wang, Peng Zheng, Mengchen Yang, Yongjun Li, Genshan Ma, Yuyu Yao

**Affiliations:** 1 Department of Cardiology, Zhongda Hospital, School of Medicine, Southeast University, Nanjing, Jiangsu, China

**Keywords:** adipose tissue, de novo lipogenesis, glycolysis, hif1α, hypertriglyceridemia, serpina3c

## Abstract

Adipose tissue dysfunction leads to abnormal lipid metabolism and high inflammation levels. This research aims to explore the role of Serpina3c, which is highly expressed in adipocytes, in obesity-related hypertriglyceridemia and metaflammation. Serpina3c global knockout (KO) mice, adipocyte-specific Serpina3c overexpressing mice, Serpina3c knockdown (KD) mice, and hypoxia-inducible factor 1 alpha (Hif1α) KD mice were fed a high-fat diet (HFD) for 16 weeks to generate obesity-related hypertriglyceridemia mice models. In the present study, Serpina3c KO mice and adipocyte-specific Serpina3c KD mice exhibited more severe obesity-related hypertriglyceridemia and metaflammation under HFD conditions. Serpina3c KO epididymal white adipose tissue (eWAT) primary stromal vascular fraction (SVF)-derived adipocytes exhibited higher lipid (triglyceride and non-esterified fatty acid) levels and higher fatty acid synthase expression after palmitic acid (PA) stimulation. Adipocyte-specific Serpina3c overexpression in KO mice prevented the KO group phenotype. The RNA-seq and *in vitro* validation revealed that Hif1α and the glycolysis pathways were up-regulated in Serpina3c KD adipocytes, which were all validated by *in vitro* and *in vivo* reverse experiments. Co-immunoprecipitation (co-IP) provided evidence that Serpina3c bound nuclear factor erythroid 2-related factor 2 (Nrf2) regulates Hif1α. Nrf2 KD reduced Hif1α and Fasn expression, decreased lipid content, and reduced the extracellular acidification rate in Serpina3c KO adipocytes. Metabolomics revealed that lactic acid (LD) levels in eWAT were responsible for adipose-associated macrophage inflammation. In summary, Serpina3c inhibits the Hif1α-glycolysis pathway and reduces de novo lipogenesis (DNL) and LD secretion in adipocytes by binding to Nrf2, thereby improving HFD-induced lipid metabolism disorders and alleviating adipose tissue macrophage inflammation.

## Introduction

Obesity is a serious threat to public health and is recognized by the World Health Organization as a chronic disease [1–3]. The development of obesity is known to exacerbate adipose tissue metaflammation [4–6]. The limited ability of human peripheral white adipose tissue (WAT) to store excess lipids is related to metabolism disorders [7,8]. When adipocytes can no longer expand, triglyceride (TG) overflow occurs, leading to hypertriglyceridemia and ectopic lipid accumulation in organs such as the liver and skeletal muscles [9]. The intricate balance between TG hydrolysis in WAT and de novo lipogenesis (DNL) is crucial for maintaining lipid homeostasis. Dysregulation of these processes can result in elevated levels of non-esterified fatty acids (NEFAs), increased hepatic TG synthesis, and consequently, severe hypertriglyceridemia [10]. Therefore, WAT dysfunction stands as a key determinant of blood lipid abnormalities, underscoring the need to understand the molecular mechanisms underlying lipid metabolism and adipose tissue inflammation [11].

The serine protease inhibitor (serpin) family comprises a diverse group of proteins categorized into 16 phylogenetic branches (A–P), each with distinct functional roles [12,13]. Among these, members of the SerpinA branch are closely associated with obesity and lipid metabolism. SERPINA12, a visceral adipose tissue (VAT)-derived serine protease inhibitor, is associated with the development of obesity and inflammation in obese patients [14]. Apolipoprotein A-IV (ApoA4) can regulate the expression of SERPINA3 in hepatocytes through the nuclear receptors NR4A1 and NR1D1, thereby playing a role in anti-inflammatory and metabolic regulation [15]. This suggests a potential regulatory role for serpin family members in adipose tissue biology and metabolism. Of particular interest is Serpina3c, a mouse homolog of human SERPINA4 (encoding kallistatin). We previously reported that hepatic steatosis was aggravated in Serpina3c KO mice, accompanied by increased TG content and up-regulated lipid metabolism gene expression [16]. These findings imply a protective role for Serpina3c in modulating lipid accumulation and metabolism. Furthermore, Serpina3c has been shown to regulate adipose tissue inflammation by modulating the cathepsin G/integrin/AKT pathway [17]. Intriguingly, plasma kallistatin levels have been reported to be inversely correlated with lipid profiles in healthy African American adolescents, suggesting a potential link between kallistatin and lipid metabolism in humans [18]. However, the precise mechanisms underlying Serpina3c’s role in obesity-related hypertriglyceridemia and metaflammation remain elusive.

Currently, kallistatin/Serpina3c is primarily studied as a secreted protein for its regulation of adipocyte function [17,19]; however, it also functions as a binding protein within cells. Research has shown that kallistatin binds to KLF4 to regulate eNOS expression in endothelial cells and interacts with NR4A1 to modulate acetylation in fibroblasts [20,21]. To date, there are no reports on the study of kallistatin/Serpina3c as a binding protein within adipocytes. Given the accumulating evidence pointing to Serpina3c’s involvement in both lipid metabolism and adipose tissue inflammation, this study represents the first use of primary adipocytes to investigate the relationship between Serpina3c and adipocyte function. This study ultimately demonstrates that Serpina3c interacts with the intracellular binding protein Nrf2 in adipocytes to inhibit the Hif1α-glycolysis axis, thereby alleviating obesity-related hypertriglyceridemia and metabolic inflammation.

## Materials and methods

### Generation of the Serpina3c KO mouse model

The generation of global Serpina3c KO mice on a C57BL/6 background was accomplished by the deletion of exon 3 at the mouse Serpina3c locus on chromosome 12 using the Cre/loxP recombination system (completed by Beijing Biocytogen Co., Ltd.). The polymerase chain reaction (PCR) primers used for the Serpina3c gene are presented in (Supplementary Table S1). Genotyping information can be found in Supplementary Figure S1.

All procedures were conducted according to the National Institutes of Health Guidelines for the Care and Use of Animals and were approved by the Care of Experimental Animals Committee of Southeast University.

### Mouse model of obesity-related hypertriglyceridemia

The mice were housed under standard conditions at 22 ± 1°C and a humidity level of 50%-60% with a 12-hour light–dark cycle at the Animal Center of Southeast University. All animals were provided free access to water and free access to either normal food (Normal diet, ND) (XTCON50J, XieTong) or an HFD (XTHF60, XieTong). Eight-week-old male mice were fed an HFD for 16 weeks to produce obesity-related hypertriglyceridemia mice [22,23].

### Generation of AAV serotype 8 (AAV8)-mediated adipocyte-specific Serpina3c-overexpressing, Serpina3c knockdown, and Hif1α knockdown mouse models

The adipocyte-specific Serpina3c-overexpressing (OV) mouse model and adipocyte-specific Hif1α knockdown (KD) model were generated by the subcutaneous injection of Serpina3c gene-overexpressing plasmids or Hif1α KD plasmids with the adiponectin promoter coated by AAV8 viral particles (AAV8-Adipoq-Serpina3c/AAV8-Adipoq-shHif1α) into the inguinal WAT (iWAT) of 12-week-old Serpina3c KO male mouse background (10^12^ pfu/mouse). As a negative control, AAV8-Adipoq-NC particles were injected into the iWAT (10^12^ pfu/mouse) [24,25].

AAV8-Adipoq-shSerpina3c viral particles (or AAV8-Adipoq-NC particles) were subcutaneously injected into the iWAT of 12-week-old wild-type (WT) mice (Genechem Co., Ltd, Shanghai) to generate adipocyte-specific Serpina3c KD mice [24,25].

### Body composition and food intake analysis

The body composition of 24-week-old mice was determined using a 7.0 T micro-magnetic resonance imaging (MRI) instrument (PharmaScan, Bruker, Germany). Food intake was monitored weekly over 24 weeks, and the average daily food intake was calculated.

### Blood collection and serum metabolite measurements

After mice were fasted overnight and then anesthetized using 1.5% isoflurane, blood samples were collected through the posterior orbital venous plexus. Serum TG [A110-1-1, Nanjing Jiancheng Bioengineering Institute (NJBI)], total cholesterol (TC) (A111-1-1, NJBI), NEFA (A042-2-1, NJBI), high-density lipoprotein cholesterol (HDL-C) (A112-1-1, NJBI), low-density lipoprotein cholesterol (LDL-C) (A113-1-1, NJBI), lactic acid (LD, A019-2-1, NJBI), alanine aminotransferase (ALT) (C009-2-1, NJBI), and aspartate aminotransferase (AST) (C010-2-1, NJBI) levels were measured using appropriate kits in accordance with the manufacturer’s instructions. The serum levels of insulin were determined using an ELISA kit (E-EL-M1382, Elabscience, Wuhan).

### Tissue metabolite measurements

Mice fed an HFD for 16 weeks were euthanized at the age of 24 weeks by cervical dislocation after anesthetization with an intraperitoneal injection of sodium pentobarbital (1%, 50 mg/kg) [26]. The TG (A110-1-1, NJBI), NEFA (A042-2-1, NJBI), LD (A019-2-1, NJBI), and citrate (E-BC-K351-M, Elabscience) levels in WAT were determined using the appropriate kits.

### Hematoxylin-eosin (H&E) staining, immunohistochemistry, and immunofluorescence

Tissues (iWAT, eWAT, BAT, heart, liver, and muscle) were collected and fixed in 4% paraformaldehyde for H&E staining, immunohistochemistry, or immunofluorescence. The inflamed adipocytes were identified by the detection of Mac2-positive CLSs within eWAT. Related antibodies are listed in Supplementary Table S2.

### Oil Red O staining

OCT-embedded frozen sections (heart, liver, and muscle) were washed once in 60% isopropanol and then stained with Oil Red O solution. Confluent 3T3-L1 adipocytes or primary adipocytes were fixed in 4% paraformaldehyde and then stained with Oil Red O solution at room temperature.

### Seahorse analysis

A Seahorse XF Glycolysis Stress Test Kit (Agilent Technologies) was used to measure the extracellular acidification rate (ECAR). Briefly, 2 × 10^4^ primary adipocytes/well were seeded into a SeahorseXFe96 FluxPak culture microplate and cultured overnight. After treatment with 500 µM palmitic acid (PA) for 24 h, the culture microplate was analyzed on a Seahorse XFe96 Analyzer (as detailed in the manufacturer’s instructions). Rot/AA (0.5 μmol/L) and 2-DG (50 mmol/L) were sequentially injected into each well at the indicated time points for ECAR measurements.

### Primary stromal vascular fraction-derived adipocyte production, differentiation, and treatment

eWAT was isolated from WT and Serpina3c KO male mice at 6 weeks and digested with collagenase II (2 mg/mL, Worthington, USA) for 30–60 min at 37 °C, and then filtered and centrifuged. The SVF was then suspended in complete Dulbecco’s modified Eagle medium (DMEM). White adipogenesis induction was performed on SVF using a complete DMEM culture medium containing 0.5 mmol/L isobutylmethylxanthine (IBMX), 1 µmol/L dexamethasone, and 10 μg/mL insulin for 2 days, and then culture medium with 10 µg/mL insulin for 2 days. Finally, the cells were cultured in complete DMEM culture medium for 4 days [27]. After obtaining mature adipocytes from KO eWAT SVF, 500 µM PA containing a glycolysis inhibitor (2-DG, an inhibitor of hexokinase 2 (Hk2)); HY-13966, MedChemExpress, USA), a lactate inhibitor (GNE140 racemate, an inhibitor of lactate dehydrogenase A (Ldha); HY-100742, MCE), or recombinant Serpina3c protein was added for 24 h of stimulation.

### Bone marrow-derived macrophage production and differentiation

Mouse bone marrow (BM) was flushed from the tibias and femurs of C57BL/6 male mice (6-8 weeks old). BM suspensions were then filtered. After removing red blood cells (RBCs) by sterile lysis, the samples were washed and centrifuged and then re-suspended with a complete DMEM medium supplemented with macrophage colony-stimulating factor (20 ng/mL).

### Molecular docking and co-immunoprecipitation assays

Molecular docking was performed with the help of a specialized molecular modeling service company (PRIMARY BIOTECH, China). At least 1 mg of total protein lysate from each sample was used for the immunoprecipitation (IP) experiments. All samples were incubated with 2 µg primary antibodies overnight at 4 °C with gentle agitation. The next day, 5 µL of Protein A agarose and 5 µL of Protein G agarose were added to each sample, and the samples were incubated for 3 h at 4 °C. The agarose beads were then washed three times, and bound protein was eluted with IP buffer for subsequent immunoblotting.

### RNA sequencing

Total RNA was extracted from PA-treated Serpina3c knockdown 3T3-L1 adipocytes, PA-treated Serpina3c overexpression 3T3-L1 adipocytes, and PA-treated corresponding control 3T3-L1 adipocytes. RNA sequencing (RNA-seq) was subsequently performed following sample testing, library construction, and library quality control (Applied Protein Technology Co., Ltd., China). Bioinformatic analyses were performed using the OmicShare tools, a free online platform for data analysis (https://www.omicshare.com/tools).

### Untargeted metabolomics

Metabolomics was performed on adipose samples from mice fed an HFD for ten weeks. All analyses were performed by liquid chromatography-mass spectrometry (LC-MS) on an UltiMate 3000 UHPLC System (Thermo Fisher Scientific, USA). An Orbitrap Exploris 120 mass spectrometer was used for first and second level mass spectrometry data acquisition under the control software (Xcalibur, version 4.4, Thermo) (BIOTREE, Shanghai).

### Small interfering RNAs transfection

Nuclear factor erythroid two-related factor 2 (Nrf2) small interfering RNA (siRNA), Hif1α siRNA, and control RNA were commercially generated (Genepharm, China). The sequences of these RNAs are listed in Supplementary Table S1. Nrf2 siRNA and Hif1α siRNA were separately transfected into Serpina3c KO primary adipocytes according to the manufacturer’s instructions. Normal control (NC) groups were treated with a non-targeting control siRNA.

### Plasmid transfection procedure

Plasmid transfection was performed when a cell confluence of 60-80% was reached and was validated according to the manufacturer’s instructions (AD600075, Zetalife, USA).

### Quantitative real-time PCR (qRT-PCR)

Total RNA was isolated from tissues and cells using TRIzol reagent (Invitrogen, USA). cDNA synthesis was performed on the isolated RNA using HiScript III RT SuperMix (R323, Vazyme, China) according to the manufacturer’s protocol. The expression levels of genes of interest were then quantified by qRT-PCR (Applied Biosystems, Carlsbad) using an SYBR Green qPCR kit (Q711, Vazyme). The sequences of all primers used are available in Supplementary Table S1.

### Western blotting

The adipose tissues and cells were homogenized in lysis buffer, and concentrations were detected using a BCA protein assay kit (BL521A, Biosharp, Chongqing). Protein samples were separated by SDS-PAGE using a 4–20% Bis-Tris protein gel. The separated protein samples were then transferred to PVDF membranes for Western analyses. All antibodies used are listed in Supplementary Table S2,1.

### Patient enrollment

In total, 78 patients from a coronary artery disease (CAD) cohort screened within the Department of Cardiology, Zhongda Hospital, between March 2019 and December 2020, were included. This study was approved by the Ethics Committee of Zhongda Hospital affiliated with Southeast University (2018ZDSYLL134-P01). Inclusion criteria: (1) male or non-pregnant female aged 18–85, (2) willing to sign written informed consent. Exclusion criteria: (1) acute myocardial infarction (AMI), (2) previous coronary artery bypass grafting history, (3) acute ischemic stroke or bleeding, (4) severe liver or kidney diseases, (5) uncontrolled severe hypertension, (6) severe dyspnea, (7) recent surgery history, (8) suffering from other severe diseases and having a life expectancy less than a year, and (9) any other circumstances deemed unsuitable for participation in the study by the researchers.

All patients were divided into three groups based on their calculated body mass index (BMI): BMI < 24 (*n* = 31); 24 ≤ BMI < 28 (*n* = 28); and BMI ≥ 28 (*n* = 19). Blood samples were collected from all patients. Supplementary Table S3,1 presents the clinical and biochemical characteristics of the subjects.

Blood samples were collected using EDTA tubes. Then the samples were centrifuged promptly at 3000 rpm for 20 minutes at 4℃ to collect the upper plasma. The kallistatin levels in patient plasma samples were measured using ELISA kits (E-EL-H5550, Elabscience).

### Statistical analyses

In the process of data analysis, we utilized three software tools: SPSS 26.0, GraphPad Prism 9.5.0, and MedCalc 15.0. For continuous data, the results were presented as mean ± standard deviation. When the data simultaneously satisfied the assumptions of normal distribution and homogeneity of variance, we employed the unpaired t-test to compare differences between the two groups. For comparisons involving more than two groups, we applied one-way ANOVA, coupled with Tukey’s multiple comparisons test or the Student–Newman–Keuls test to assess statistical significance. In cases where the data only met the assumption of normal distribution but not homogeneity of variance, we utilized the unpaired t-test with Welch’s correction for comparisons between two groups and Welch’s ANOVA test for comparisons among multiple groups. For data that did not adhere to a normal distribution, we opted for the Mann–Whitney test for comparisons between two groups and the Kruskal–Wallis test for comparisons involving multiple groups. For categorical variables, we conducted comparisons using the chi-squared test. A *P*-values less than 0.05 were considered statistically significant, indicating a low probability of observing the data under the null hypothesis.

## Results

### Serpina3c expression in adipose tissue and adipocytes

Serpina3c was significantly expressed in WAT in WT mice. However, Serpina3c expression was very low in brown adipose tissue (BAT), heart, liver, kidneys, muscles, lungs, and aortas (Supplementary Figure S2A). Western blot analysis showed that Serpina3c was down-regulated in WAT (Supplementary Figure S2B-S2C) after 16 weeks of HFD feeding. GEO datasets GSE134914 downloaded from PubMed included RNA-seq results of iWAT and eWAT, from mice with 12 weeks HFD, and GSE131861 included RNA-seq results of different adipose tissue from mice with ND. Re-analysis of these two datasets validated that Serpina3c expression was down-regulated in eWAT after HFD (Supplementary Figure S2D). Consistent with this finding, qRT-PCR analysis further demonstrated a reduction in Serpina3c expression levels in eWAT of mice fed an HFD (Supplementary Figure S2E). Our study further revealed that Serpina3c was expressed at low levels in preadipocytes derived from eWAT SVF, and Serpina3c was expressed at high levels in adipocytes ( Supplementary Figure S2F). While Serpina3c expression levels were relatively low in preadipocytes, bioinformatic analysis of single-cell sequencing datasets (GSE161872 and GSE237143) showed Serpina3c was almost not expressed in other cell types ( Supplementary Figure S2G-S2J), and its expression was decreased in preadipocytes of the HFD group.

### Serpina3c deficiency exacerbated HFD-induced obesity, obesity-related hypertriglyceridemia, and metaflammation

**Figure 1:**
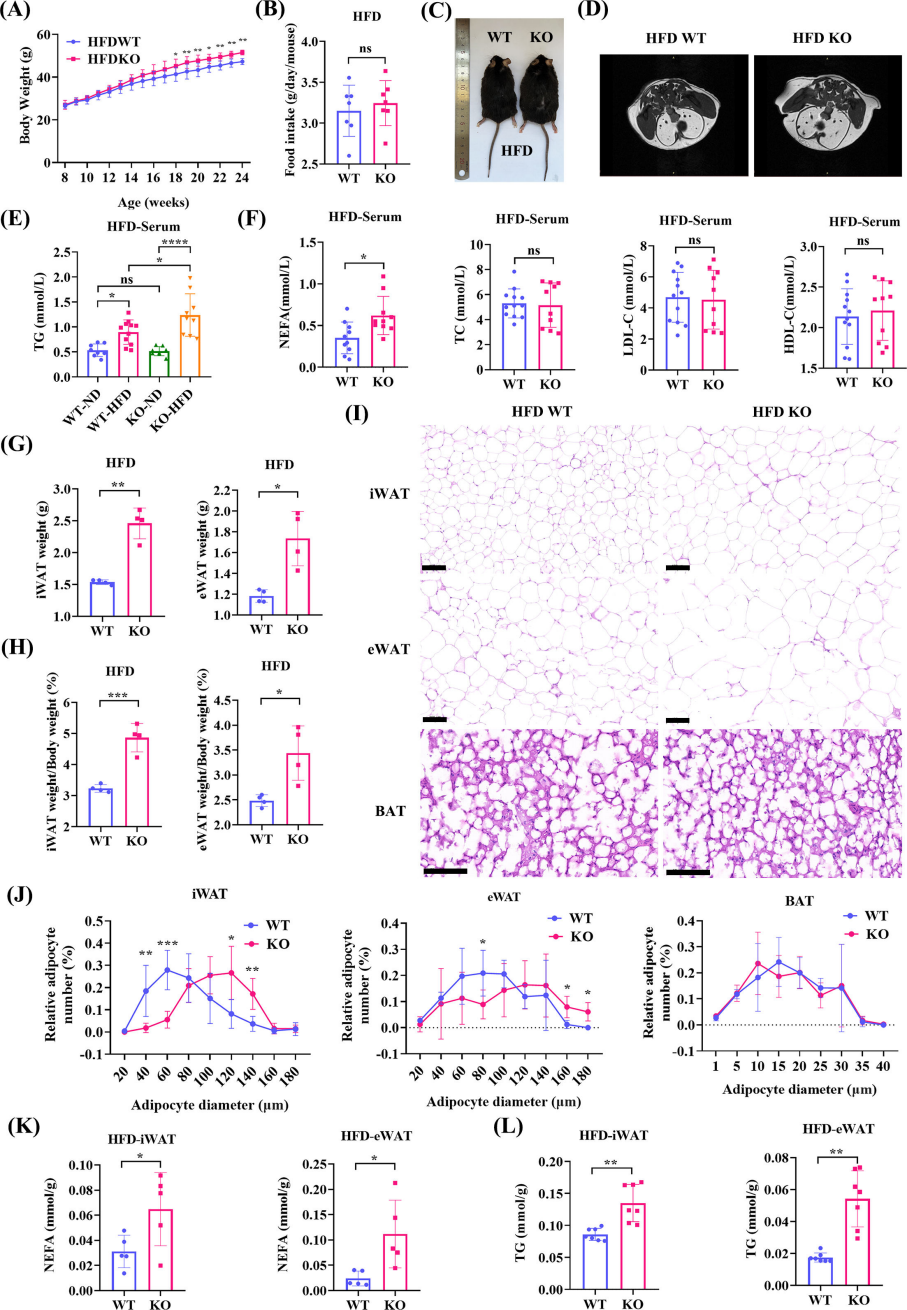
Serpina3c KO increased fat weight and hypertriglyceridemia of mice fed HFD. (**A**) Body weight of HFD-fed WT and KO mice (*n* = 10, *P*<0.05). (**B**) Food intake of WT and KO mice per day (*n* = 7, *P*=0.5615). (**C**) Morphology of WT and KO mice (*n* = 7). (**D**) Representative micro-MRI images of WT (*n* = 4) and KO (*n* = 4) mice. (**E**) Serum TG levels in ND-fed and HFD-fed WT and Serpina3c KO mice (*n* = 8, *P*<0.05). (**F**) Serum NEFA levels in WT and KO mice (*n* = 10, *P*=0.0107). Serum TC (*P*=0.8321, unpaired t-test), LDL-C (*P*=0.8335), and HDL-C (*P*=0.6352) levels in WT (*n* = 10) and KO (*n* = 10) mice. (**G**) Weight of iWAT (*P*=0.0041) and eWAT (*P*=0.0214) from WT (*n* = 4) and KO (*n* = 4) mice. (**H**) The proportion of iWAT weight to body weight (*P*=0.0005) and eWAT weight to body weight (*P*=0.0368) in WT (*n* = 4) and KO (*n* = 4) mice. (**I**) H&E staining of iWAT, eWAT, and BAT from WT (*n* = 4) and KO (*n* = 4) mice (bar = 100 μm). (**J**) Adipocyte cell size in iWAT, eWAT, and BAT from WT and KO mice (*n* = 4–5, *P*<0.05). (**K**) NEFA levels were measured in iWAT (*n* = 5, *P*=0.0454) and eWAT (*n* = 5, *P*=0.0409) from WT and KO mice. (**L**) TG levels were measured in iWAT (*n* = 7, *P*=0.0012) and eWAT (*n* = 7, *P*=0.0013) from WT and KO mice. Values are presented as the mean ± SD. ns: not significant, *P < 0.05, **P < 0.01, ***P < 0.001, ****P < 0.0001.

The mean body weight of Serpina3c KO mice was increased compared with WT mice after groups were fed an HFD for more than ten weeks (Figure 1A). After 16 weeks of HFD feeding, food intake was similar in both the WT and KO groups (Figure 1B). The Serpina3c KO mice that were fed an HFD were also significantly fatter compared with WT mice (Figure 1C). The increased body weight of Serpina3c KO mice was associated with fat mass, as presented using Micro-MRI (Figure 1D).

**Figure 2:**
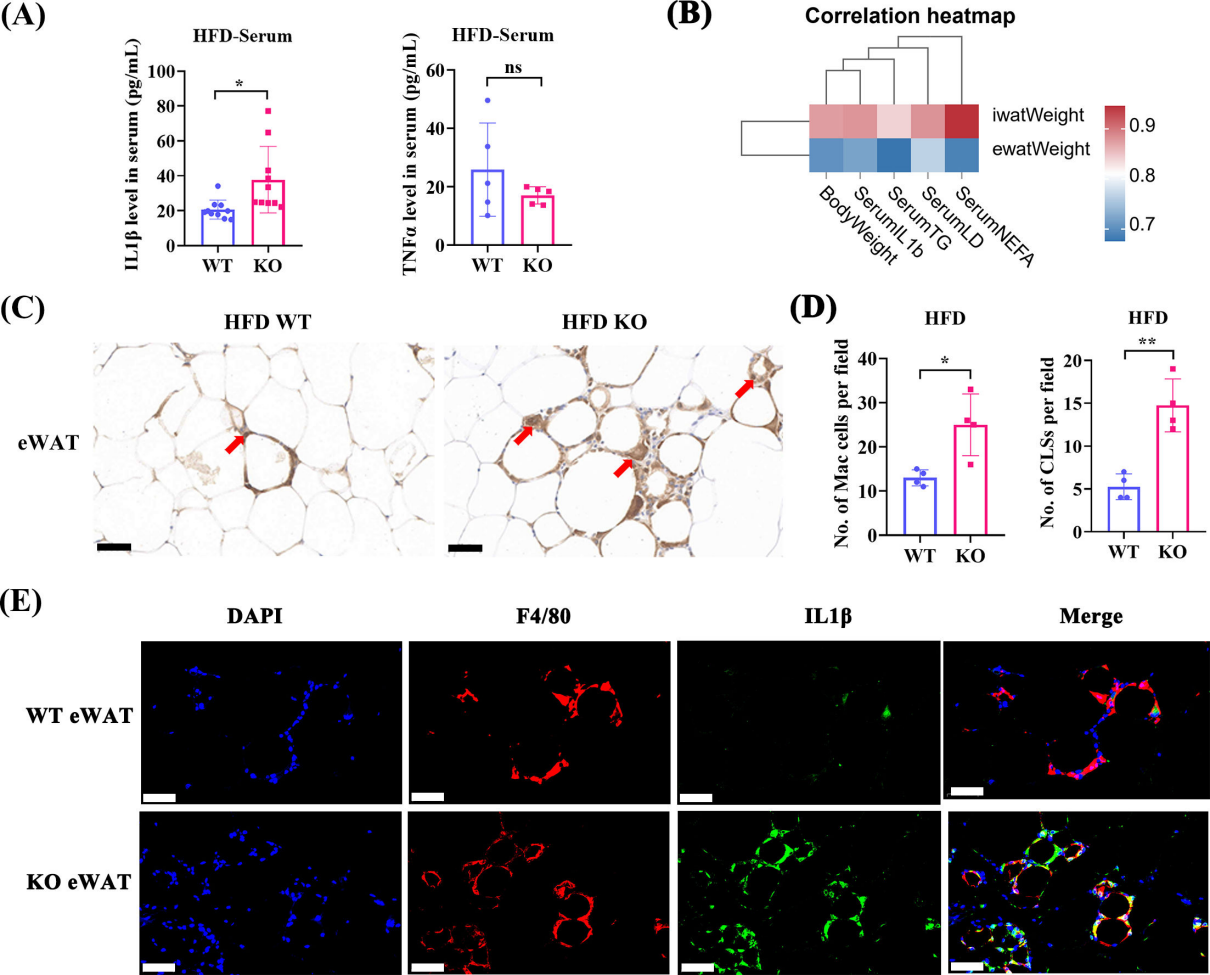
Serpina3c deletion exacerbated HFD-induced systemic inflammation. (**A**) Serum IL1β levels in HFD-fed WT and KO mice (*n* = 10, *P*=0.0137). Serum TNFα levels in HFD-fed WT and KO mice (*n* = 5, *P*=0.2581). (**B**) Pearson analysis exhibited that iWAT weight was positively associated with body weight (*r* = 0.879, *P*=0.004), serum TG (*r* = 0.843, *P*=0.009), and serum IL 1β (*r* = 0.786, *P*=0.021). eWAT weight was positively associated with body weight (*r* = 0.705, *P*=0.051) and serum TG (*r* = 0.678, *P*=0.064). (**C,D**) Coronal structure and quantification of eWAT from HFD-fed WT and KO mice (*n* = 4, *P*<0.05). (**E**) Immunofluorescence staining of F4/80^+^, IL1β, and DAPI in eWAT (*n* = 4) from HFD-fed WT and KO mice (bar = 50 μm). Data are presented as the mean ± SD. ns: not significant, *P < 0.05, **P < 0.01, ***P < 0.001, ****P < 0.0001.

After 16 weeks of HFD feeding, lipid profile analysis revealed a notable increase in serum TG and NEFA (Figure 1E–1F) levels in KO mice (compared with WT mice), but no significant change in serum TC, LDL-C, or HDL-C levels (Figure 1F). The iWAT and eWAT depots isolated from KO mice were heavier than the corresponding samples isolated from WT mice (Figure 1G–1H). iWAT and eWAT samples from Serpina3c KO mice exhibited white adipocyte hypertrophy compared with the corresponding samples from WT mice (Figure 1I–1J). However, H&E staining showed no morphological changes in BAT adipocytes in the HFD-fed WT and KO groups (Figure 1I–1J). Consistently, lipid accumulation in iWAT and eWAT was increased in Serpina3c KO mice (compared with WT mice) (Figure 1K–1L). In Serpina3c KO mice, serum IL1β levels were significantly increased compared with WT mice (Figure 2A). Serum TNFα levels were similar between KO and WT mice (Figure 2A). Pearson analysis exhibited that iWAT weight and eWAT weight were both positively associated with body weight, TG, and IL 1β in serum (Figure 2B). This result suggests that Serpina3c deficiency in adipose tissue may be a primary cause of dyslipidemia and inflammation.

Metaflammation accompanied by adipose metabolic disorders induced by HFD is considered to be mediated by macrophage accumulation in adipose tissues. eWAT samples from Serpina3c KO mice exhibited additional crown-like structures (CLSs) compared with the samples from WT mice (Figure 2C–2D). Immunofluorescence analysis of adipose sections revealed that co-localization of F4/80^+^ and IL1β^+^ macrophages was increased in KO mice (Figure 2E).

We also simultaneously observed the effects of Serpina3c KO on lipid droplet infiltration in other organs. Pronounced hepatocyte ballooning and vacuolation and the increased accumulation of lipid droplets in the liver were observed in HFD-fed Serpina3c KO mice (Supplementary Figure S3A–3C). However, no significant differences in serum ALT and AST concentrations were observed between KO and WT mice (Supplementary Figure S3D–S3E). No significant difference in the morphology of cardiomyocytes (Supplementary Figure S3F) or skeletal muscle cells was observed (Figure S3G). However, Oil Red O staining revealed an increased accumulation of lipid droplets in the heart (Supplementary Figure S3H) and muscle (Supplementary Figure S3I) of KO mice.

**Figure 3:**
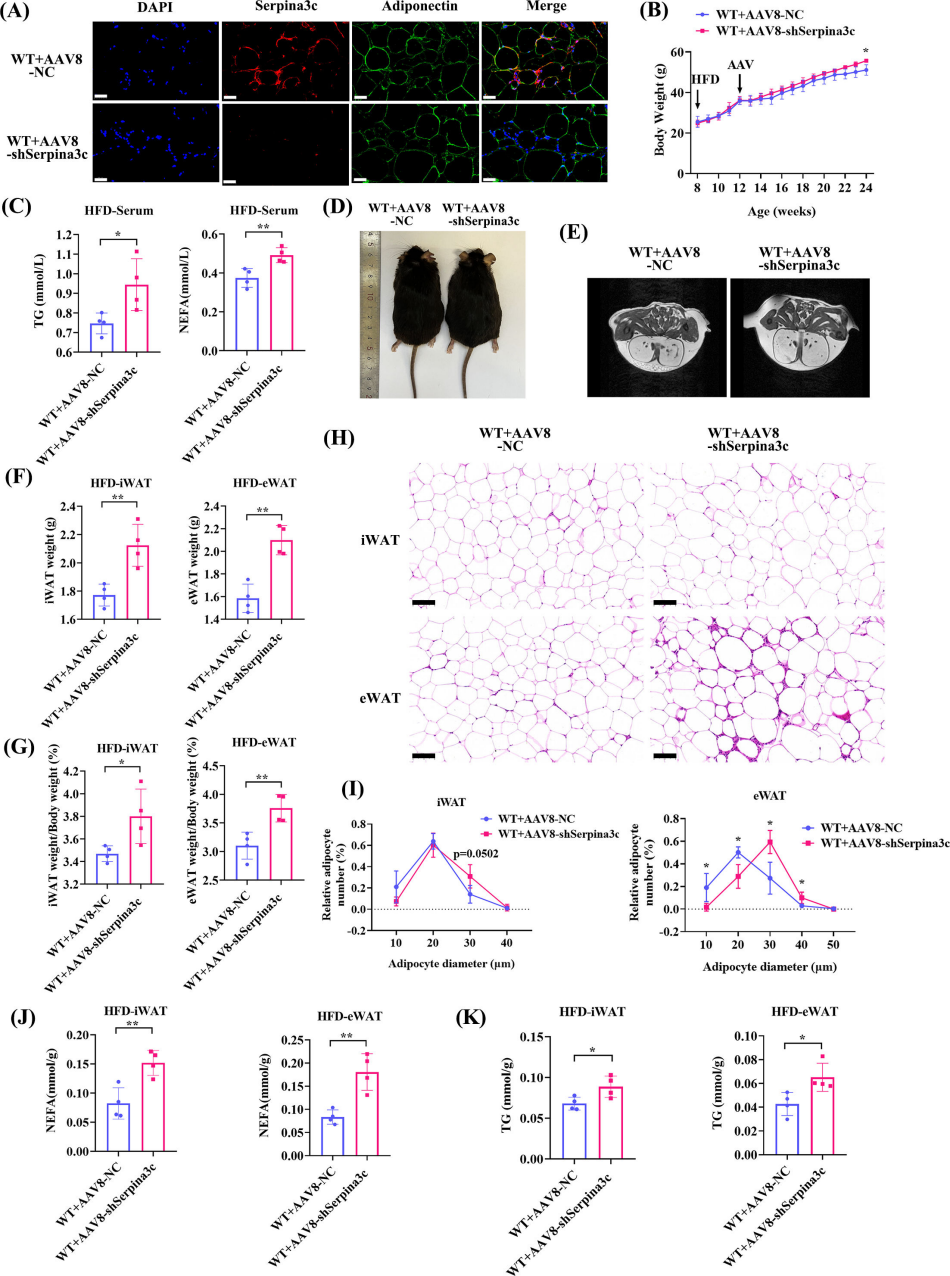
AAV8-mediated adipocyte-specific knockdown of Serpina3c promoted fat hypertrophy and hyperlipidemia in HFD mice. (**A**) Immunofluorescent staining of Serpina3c, adiponectin, and DAPI in eWAT from HFD-fed AAV8-Adipoq-NC and AAV8-Adipoq-shSerpina3c mice (*n* = 3, bar = 50 μm). (**B**) Mice receiving AAV8-Adipoq-shSerpina3c (*n* = 5) had increased body weight after HFD feeding than AAV8-Adipoq-NC (*n* = 4) mice (*P*<0.05). (**C**) Serum TG levels (*n* = 4, *P*=0.0323) and NEFA levels (*n* = 4, *P*=0.0089) of HFD-fed AAV8-Adipoq-shSerpina3c mice were increased compared with AAV8-Adipoq-NC mice. (**D**) Mice receiving AAV8-Adipoq-shSerpina3c were fatter after HFD feeding (*n* = 3) than AAV8-Adipoq-NC mice. (**E**) Mice receiving AAV8-Adipoq-shSerpina3c had increased WAT area after HFD feeding as evaluated by micro-MRI (*n* = 3) compared with AAV8-Adipoq-NC mice. (**F**) iWAT (*n* = 4, *P*=0.0057) and eWAT (*n* = 4, *P*=0.0013) weights of HFD-fed AAV8-Adipoq-shSerpina3c mice were increased compared with AAV8-Adipoq-NC mice. (**G**) The proportions of iWAT weight to body weight (*n* = 4, *P*=0.0388) and eWAT weight to body weight (*n* = 4, *P*=0.0081) were both increased in HFD-fed AAV8-Adipoq-shSerpina3c mice than AAV8-Adipoq-NC mice. (**H**) H&E staining exhibited more hypertrophic adipocytes in iWAT and eWAT of HFD-fed AAV8-Adipoq-shSerpina3c mice than AAV8-Adipoq-NC mice (*n* = 4, bar = 100 μm). (**I**) Adipocyte diameter of HFD-fed AAV8-Adipoq-NC and AAV8-Adipoq-shSerpina3c mice eWAT (*n* = 4, *P*<0.05) and iWAT (*n* = 4, *P*=0.0502). (**J**) NEFA levels were increased in iWAT (*n* = 4, *P*=0.0068) and eWAT (*n* = 4, *P*=0.0038) of HFD-fed AAV8-Adipoq-shSerpina3c mice than AAV8-Adipoq-NC. (**K**) TG levels measured in iWAT (*n* = 4, *P*=0.0351) and eWAT (*n* = 4, *P*=0.0264) of HFD-fed AAV8-Adipoq-shSerpina3c was increased compared with AAV8-Adipoq-NC. Values are presented as the mean ± SD. ns: not significant, *P < 0.05, **P < 0.01, ***P < 0.001, ****P < 0.0001.

**Figure 4:**
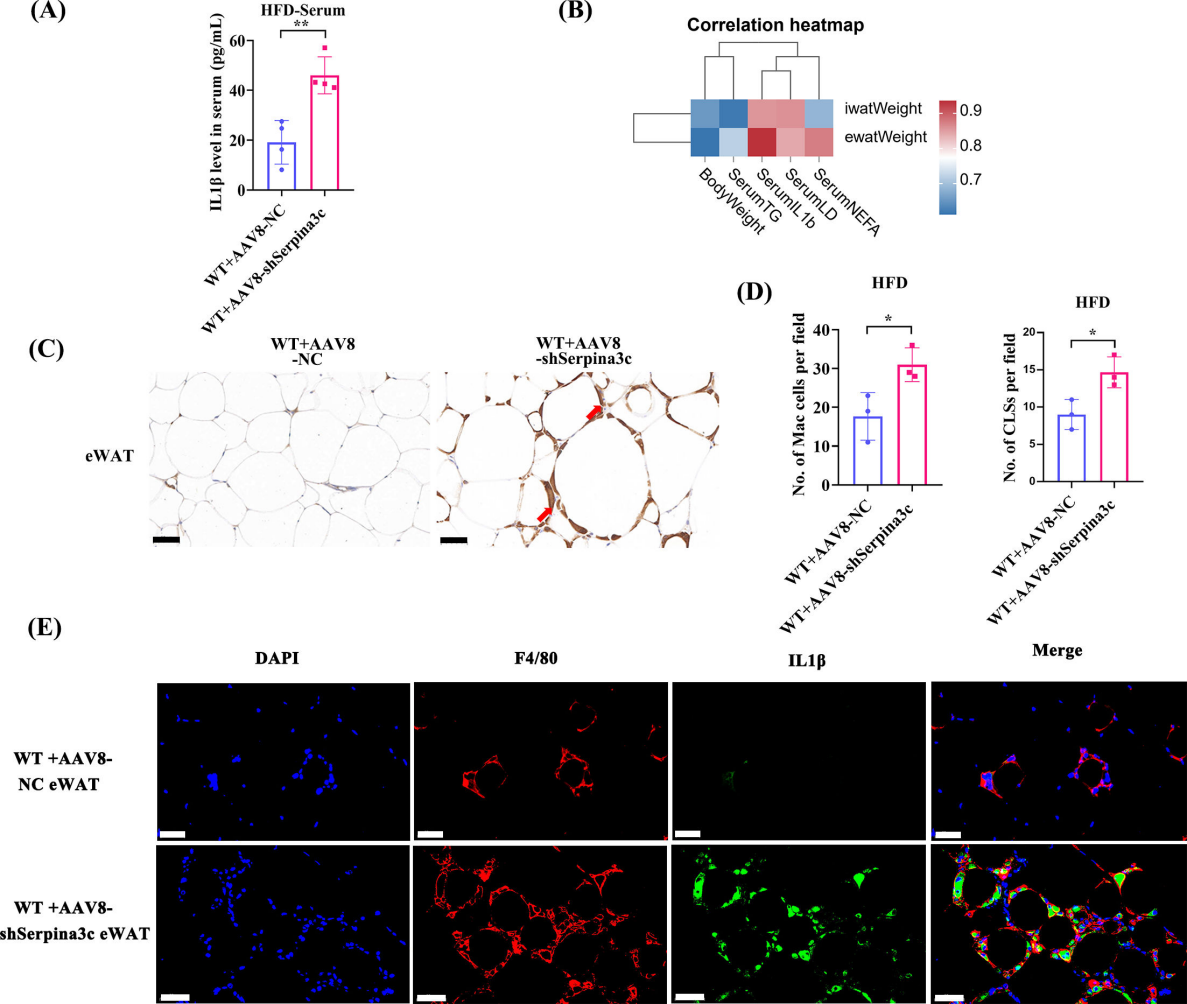
AAV8-mediated adipose-specific knockdown of Serpina3c increased HFD-fed metaflammation. (**A**) Serum IL1β levels in HFD-fed AAV8-Adipoq-NC and AAV8-Adipoq-shSerpina3c mice (*n* = 4, *P*=0.0034). (**B**) Pearson analysis exhibited that iWAT weight was positively associated with body weight (*r* = 0.656, *P*=0.078), serum TG (*r* = 0.620, *P*=0.101), and serum IL 1β (*r* = 0.860, *P*=0.006). eWAT weight was positively associated with body weight (*r* = 0.615, *P*=0.105) and serum TG (*r* = 0.721, *P*=0.044). (**C,D**) Coronal structure and quantification were increased in eWAT in HFD-fed AAV8-Adipoq-shSerpina3c mice (*n* = 4, *P*<0.05). (**E**) Immunofluorescence staining of Serpina3c, F4/80^+^, IL 1β, and DAPI in eWAT from HFD-fed AAV8-Adipoq-NC and AAV8-Adipoq-shSerpina3c mice (*n* = 3, *P*=0.0034) (bar = 50 μm). The above results suggested that AAV8-mediated adipocyte-specific Serpina3c KD exacerbated HFD-induced obesity and adipocyte hypertrophy in mice. Data are presented as mean ± SD. ns: not significant, * *P* < 0.05, **P < 0.01 compared with control group.

To further confirm that Serpina3c in adipocytes primarily regulated lipid metabolism, we generated AAV-mediated adipocyte-specific Serpina3c KD mice and established an obesity-related hypertriglyceridemia model. Crucially, the results of WAT samples from mice treated with AAV8-Adipoq-shSerpina3c were consistent with the results of WAT samples from Serpina3c global KO mice (Figures 3 and 4, Supplementary Figure S4). The aforementioned experiments further demonstrated that Serpina3c deficiency in adipocytes led to a lipid metabolism disorder.

### Deletion of Serpina3c in adipocytes activates the Hif1α pathway, resulting in an increase in glycolysis and DNL

**Figure 5:**
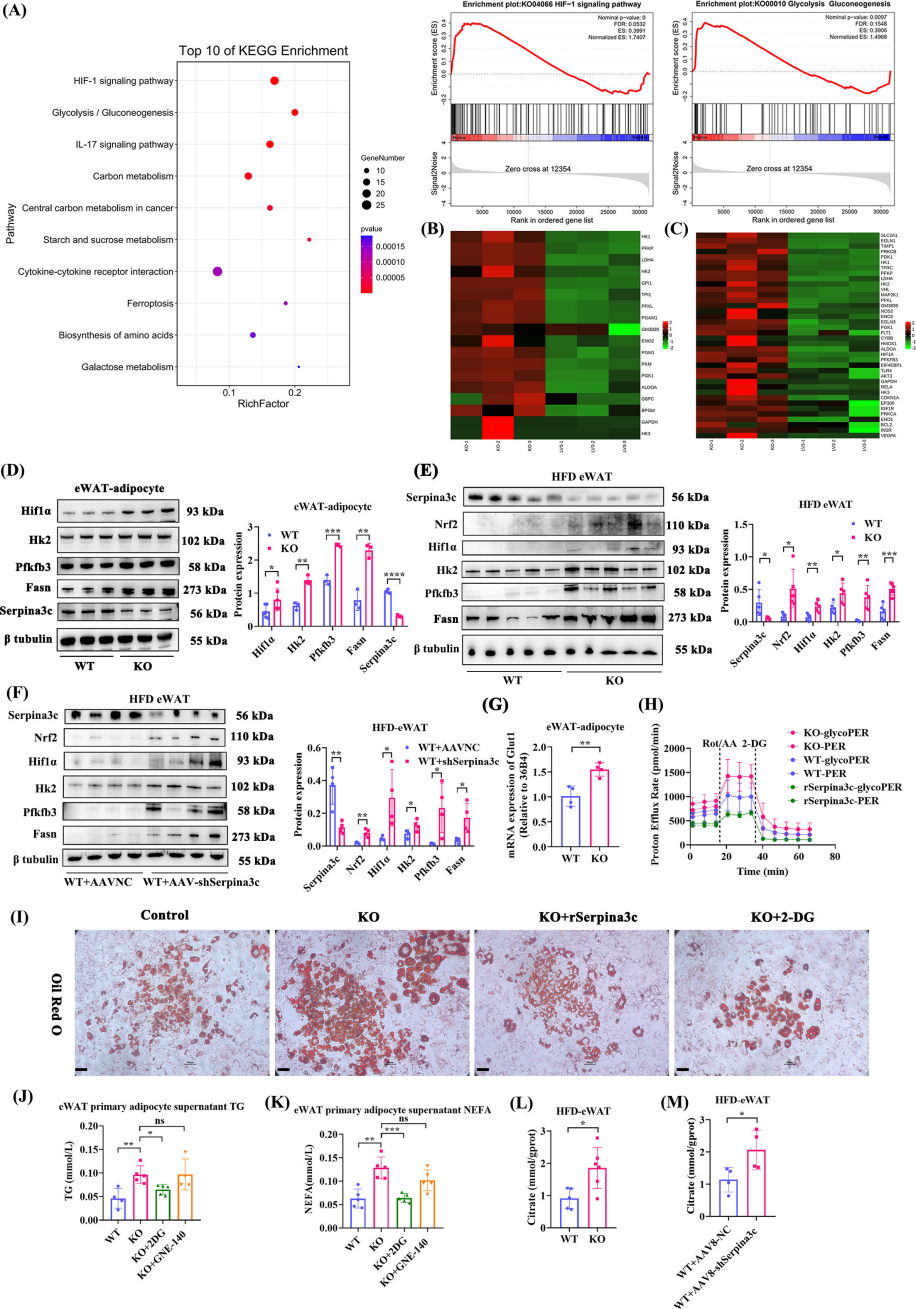
Serpina3c KO promoted the activation of Hif1α, glycolysis, and fatty acid synthesis pathways. (**A**) The top ten KEGG pathways enhanced in PA-induced Serpina3c KD 3T3-L1 adipocytes compared with NC adipocytes (*n* = 3, 500 µM PA for 24 h), and the GSEA results of Hif1α and glycolysis. (**B-C**) Heat map of RNA-seq in PA-induced Serpina3c KD 3T3-L1 adipocytes. (**D**) Protein expression of Hif1α (*P*=0.044), Hk2 (*P*=0.0018), Pfkfb3 (*P*=0.0003), Fasn (*P*=0.0021), and Serpina3c (*P*<0.0001) in PA-induced WT and KO primary adipocyte (*n* = 3–6). (**E**) Protein expression of Serpina3c (*P*=0.0289), Nrf2 (*P*=0.0131), Hif1α (*P*=0.00484), Hk2 (*P*=0.0235), Pfkfb3 (*P*=0.0028), Fasn (*P*=0.0009), and β-tubulin in eWAT from HFD-fed WT and KO mice (*n* = 5). (**F**) Protein expression of Serpina3c (*P*=0.0052), Nrf2 (*P*=0.0022), Hif1α (*P*=0.0282), Hk2 (*P*=0.0469), Pfkfb3 (*P*=0.0283), Fasn (*P*=0.0235), and β-tubulin in eWAT from HFD-fed WT+AAVNC and WT+AAV-shSerpina3c mice (*n* = 4). (**G**) mRNA expression of Glut1 in PA-induced WT and KO primary adipocyte (*n* = 4, *P*=0.0045). (**H**) Seahorse glycolysis stress test with the sequential addition of Rot/AA and 2-DG in PA-induced WT, KO, and recombinant Serpina3c protein-treated primary adipocyte (*n* = 4). (**I**) Oil Red O staining of adipocytes WT, KO, KO+rSerpina3c and KO+2DG (*n* = 5, bar = 50 μm). (**J-K**) TG (*P*<0.05) and NEFA (*P*<0.001) content in primary adipocyte culture supernatant in the PA-induced WT, KO, KO+2DG, and KO+GNE140 racemate groups (*n* = 4–5). (**L**) Citrate content measured in eWAT from HFD-fed WT and KO mice (*n* = 5–6, *P*=0.0151). (**M**) Citrate content measured in eWAT from HFD-fed AAV8-adipoq-NC and AAV8-adipoq-shSerpina3c mice (*n* = 4, *P*=0.0431). Data are presented as the mean ± SD. ns: not significant, *P < 0.05, **P < 0.01, ***P < 0.001, ****P < 0.0001 compared with the control group.

Comparative Kyoto Encyclopedia of Genes and Genomes (KEGG) pathway analysis of the RNA-seq results revealed that the Hif1 and glycolysis signaling pathways were the top two pathways up-regulated in the Serpina3c KD group compared with the control group (Figure 5A–5C). The RNA-seq results showed that the expression of the Hif1α pathway and glycolysis pathway-related molecules was prevented in Serpina3c OV 3T3-L1 adipocytes (Supplementary Figure S5A). In agreement with the above results, the expression levels of glycolytic enzymes, such as Hk2, 6-phosphofructo-2-kinase/fructose-2,6-bisphosphatase 3 (Pfkfb3), Ldha, and Hif1α, their common regulator, were increased in adipocytes derived from the KO eWAT SVF compared with the WT group after PA stimulation (Figure 5D, Supplementary Figure S5B). The expression levels of Hif1α, Hk2, and Pfkfb3 were also increased in KO eWAT (Figure 5E). Similarly, in *ex vivo* experiments, the expression of Hif1α, Hk2, and Pfkfb3 in the eWAT of AAV8-shSerpina3c mice was increased compared with that of AAVNC mice (Figure 5F). Further analysis of metabolic enzymes indicated an increase in glucose transporter-1 (Glut-1) expression (Figure 5G). These results suggest that PA-stimulated KO adipocytes exhibited a highly glycolytic metabolic profile. The glycolysis rate assay was used to quantify the proton efflux rate (PER) and the GlycoPER (Figure 5H). The %PER from glycolysis represents the contribution of the glycolytic pathway to total extracellular acidification. For the three groups tested (WT adipocytes, Serpina3c KO adipocytes, and Serpina3c KO adipocytes treated with recombinant Serpina3c protein), the contribution of glycolysis represented about 90% of the total acidification observed (Supplementary Figure S5D). As shown in Supplementary Figure S5E-S5F, basal glycolysis and compensatory glycolysis were increased in Serpina3c KO adipocytes compared with the other groups. The basal mitoOCR/glycoPER ratio was also increased in Serpina3c KO adipocytes compared with Serpina3c KO adipocytes treated with recombinant Serpina3c protein (Supplementary Figure S5G). Oil Red O staining confirmed that the PA-stimulated primary adipocytes obtained from Serpina3c KO mice eWAT exhibited a significantly higher lipid content than control adipocytes (Figure 5I). The TG content and NEFA content were both increased in supernatant from PA-stimulated KO adipocytes (Figure 5J–5K). We also observed increased levels of citrate, an intermediate between glucose metabolism and DNL, in the eWAT of HFD-fed KO and AAV8-Adipoq-shSerpina3c mice (Figure 5L–5M), demonstrating that enhanced glycolysis could facilitate DNL. The expression levels of fatty acid synthase (Fasn) protein, a key enzyme in fatty acid synthesis, were also increased in KO adipocytes (Figure 5D) and KO or AAV8-shSerpina3c eWAT (Figure 5E–5F). Adipose triglyceride lipase (ATGL) expression was increased in KO eWAT-derived adipocytes compared with WT adipocytes (Supplementary Figure S5C).

**Figure 6:**
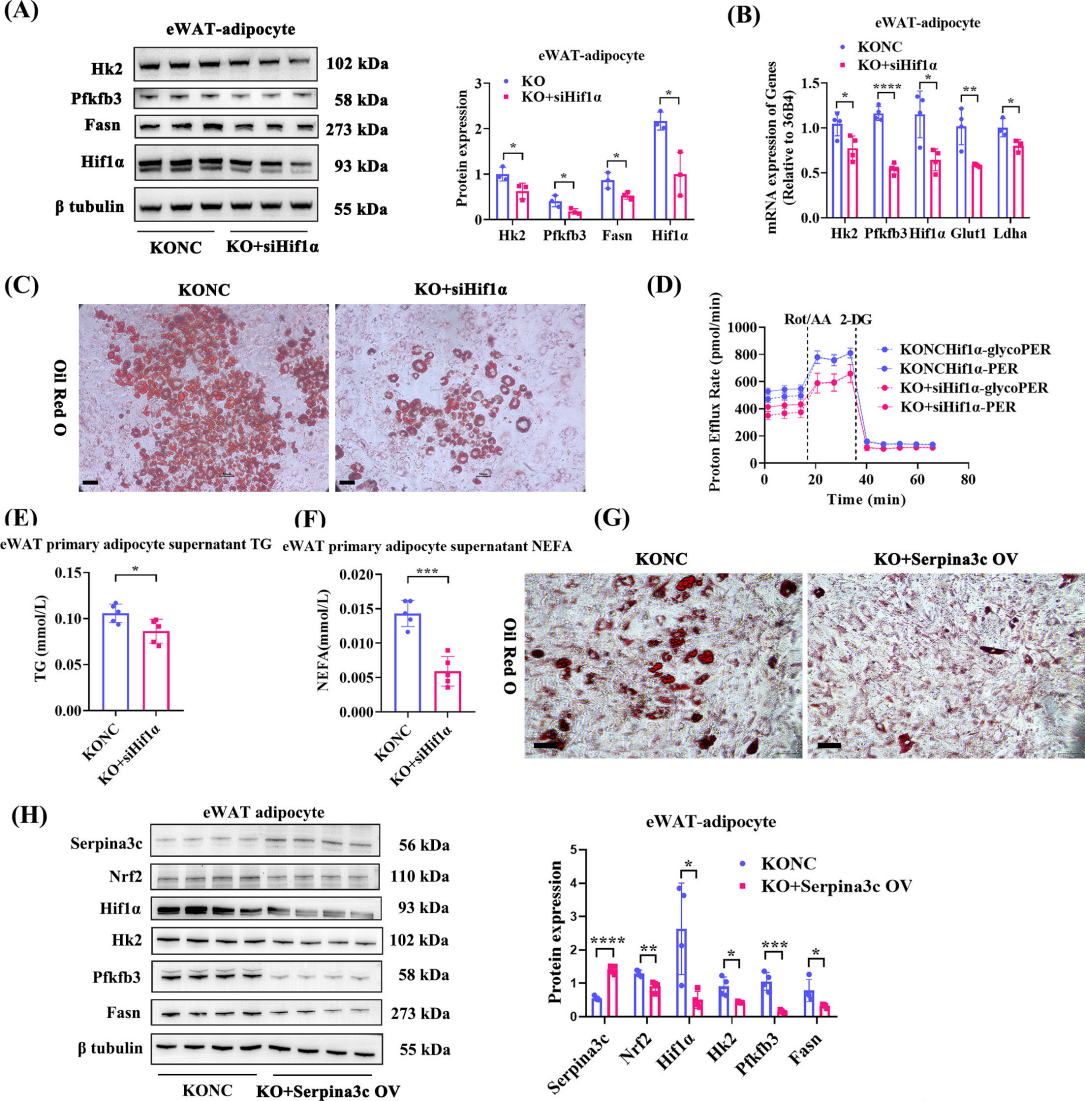
Hif1α knockdown or Serpina3c overexpression alleviated lipid accumulation by inhibiting glycolysis. (**A**) Protein expression of Hk2 (*P*=0.0486), Pfkfb3 (*P*=0.0469), Fasn (*P*=0.0397), and Hif1α (*P*=0.0178) in KO and Hif1α KD primary adipocyte (*n* = 3). (**B**) mRNA expression of Hk2 (*P*=0.0285), Pfkfb3 (*P*<0.0001), Hif1α (*P*=0.0122), Glut1 (*P*=0.0055), and Ldha (*P*=0.0462) in KO and Hif1α KD primary adipocyte (*n* = 3–4). (**C**) Oil Red O staining in PA-induced KO and Hif1α KD adipocytes (*n* = 6, bar = 50 μm). (**D**) Seahorse glycolysis stress test with the sequential addition of Rot/AA and 2-DG in PA-induced KO and Hif1α KD primary adipocyte (*n* = 4). (**E-F**) TG (*P*=0.0297) and NEFA (*P*=0.0002) content in adipocyte culture supernatant in the PA-induced NC and Hif1α KD groups (*n* = 5). (**G**) Oil Red O staining in PA-induced KO adipocytes and Serpina3c OV adipocytes in KO background (*n* = 4, bar = 50 μm). (**H**) Protein expression of Serpina3c (*P*<0.0001), Nrf2 (*P*=0.0047), Hif1α (*P*=0.0222), Hk2 (*P*=0.0162), Pfkfb3 (*P*=0.0006), Fasn (*P*=0.0326), and β-tubulin in PA-treated KO and Serpina3c OV adipocyte-derived from SVF in KO background (*n* = 4). Data are presented as the mean ± SD. ns: not significant, *P < 0.05, **P < 0.01, ***P < 0.001, ****P < 0.0001 compared with the control group.

Hif1α knockdown adipocytes were generated by siRNA on a Serpina3c KO eWAT SVF-derived adipocytes background to further investigate the role of Hif1α (Figure 6A–6B). Oil Red O staining revealed reduced lipid droplet accumulation in both the Hif1α KD group (Figure 6C) and the group treated with 2-DG (Figure 5I) compared with the control group. Using a glycolytic stress assay, we found that the Hif1α KD group also exhibited lower glycoPER/PER after PA stimulation compared with the control group (Figure 6D). Again, the contribution of glycolysis represented about 90% of the total acidification observed (Supplementary Figure S5H). Basal glycolysis and the compensatory glycolysis levels in Hif1α knockdown adipocytes were reduced compared with NC adipocytes (Supplementary Figure S5I-S5J). The basal mitoOCR/glycoPER ratio in Hif1α knockdown adipocytes was increased compared with NC adipocytes (Supplementary Figure S5K). The TG content and NEFA content were also consistently decreased in both the Hif1α knockdown (Figure 6E–6F) and 2-DG treatment groups (Figure 5J–5K) compared with the control group. Serpina3c overexpression in KO eWAT SVF-derived adipocytes also showed similar results (Figure 6G–6H).

### Serpina3c down-regulation leads to an enhancement of glycolysis and DNL via the up-regulation of Hif1α transcription, which is facilitated by increased Nrf2 expression

RNA-seq indicated the important role of the Hif1α pathway in the downstream regulation of Serpina3c (Figure 5A). The expression of Nrf2 and Hif1α was increased in Serpina3c KO adipocytes (Supplementary Figure S6A-S6B). Previous research reported that Nrf2 was a transcription factor of Hif1α. Thus, we hypothesized that Serpina3c might regulate Nrf2. Molecular docking of Nrf2 and Serpina3c was performed *in silico* to investigate the possibility of an interaction between Nrf2 and Serpina3c proteins, and energy optimization of the Nrf2-Serpina3c complex structure was performed using the NAMD program (Supplementary Figure S6C). The postulated interaction between Serpina3c and Nrf2 was also confirmed in mature adipocytes derived from WT eWAT SVF and KO eWAT SVF using a co-IP assay (Supplementary Figure S6D).

Our results reveal that Nrf2 knockdown by siRNA in Serpina3c KO adipocytes reduced the expression levels of Hif1α (Supplementary Figure S6E-S6F), glycolytic-related genes, and Fasn (Supplementary Figure S6G) compared with KO eWAT SVF-derived cells. Seahorse analysis demonstrated that Nrf2 knockdown led to a lower PER and glycoPER after PA stimulation (Supplementary Figure S6H). In addition, Oil Red O staining revealed a decrease in lipid droplet accumulation in the Nrf2 knockdown group on a Serpina3c KO background (Supplementary Figure S6I). TG and NEFA contents were significantly lower (Supplmentary Figure S6J-S6K) compared with the control group.

### Both adipocyte-specific Serpina3c overexpression and Hif1α knockdown can prevent the phenotypic changes observed in Serpina3c KO mice

**Figure 7:**
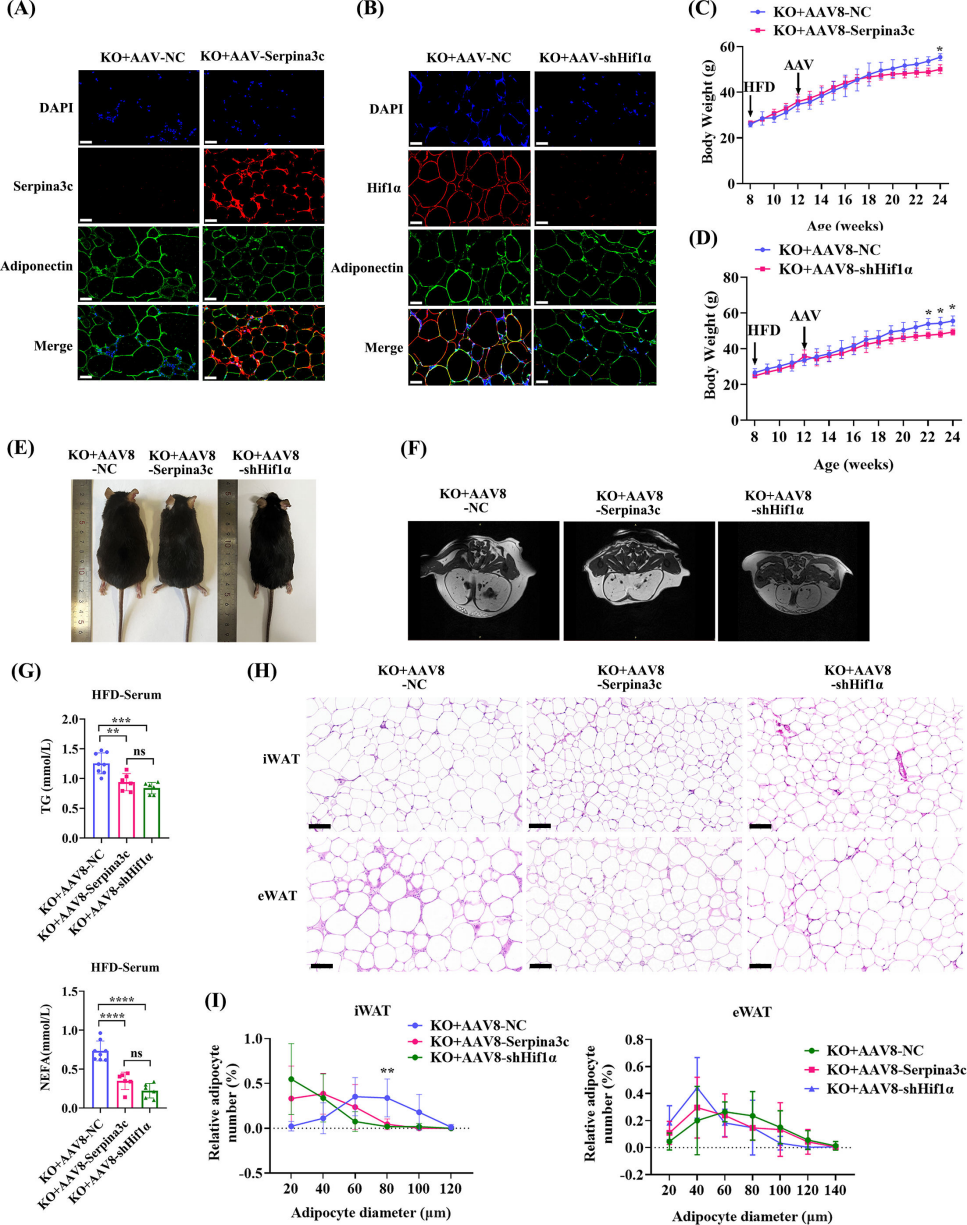
AAV8-mediated adipose-specific overexpression of Serpina3c or knockdown of Hif1α reduced HFD-fed body weight. (**A**) Immunofluorescent staining of Serpina3c, adiponectin, and DAPI in the eWAT of HFD-fed AAV8-Adipoq-NC and AAV8-Adipoq-Serpina3c mice (*n* = 3, bar = 50 μm). (**B**) Immunofluorescent staining of Hif1α, adiponectin, and DAPI in the eWAT of HFD-fed AAV8-Adipoq-NC and AAV8-Adipoq-shHif1α mice (*n* = 3, bar = 50 μm). (**C**) Body weight of HFD-fed AAV8-Adipoq-NC and AAV8-Adipoq-Serpina3c mice (*n* = 5–6) (*P*<0.05). (**D**) Body weight of HFD-fed AAV8-Adipoq-NC and AAV8-Adipoq-shHif1α (*n* = 5–6) mice, (*P*<0.05). (**E**) Morphology of HFD-fed AAV8-Adipoq-NC, AAV8-Adipoq-Serpina3c, and AAV8-Adipoq-shHif1α mice (*n* = 3). (**F**) Mice receiving AAV8-Adipoq-Serpina3c or AAV8-Adipoq-shHif1α had induced WAT area after HFD feeding compared with AAV8-Adipoq-NC mice as evaluated by Micro-MRI (*n* = 3). (**G**) Serum TG (*P*<0.001) and NEFA (*P*<0.0001) levels in HFD-fed AAV8-Adipoq-NC, AAV8-Adipoq-Serpina3c, and AAV8-Adipoq-shHif1α mice (*n* = 6–8). (**H**) H&E staining of iWAT (*n* = 4) and eWAT (*n* = 5) from three groups (bar = 100 μm). (**I**) Adipocyte cell size in iWAT (*n* = 4) and eWAT (*n* = 5) from three groups (*P*<0.01). Data are presented as the mean ± SD. ns: not significant, *P < 0.05, **P < 0.01, ***P < 0.001 compared with the control group.

Serpina3c was overexpressed in the eWAT of mice treated with AAV8-Adipoq-Serpina3c (Figure 7A). In HFD-fed mice, the observed weight gain in AAV8-Adipoq-Serpina3c mice was lower than that in AAV8-Adipoq-NC mice (Figure 7C). The body composition analysis using micro-MRI also revealed that the fat mass was reduced in AAV8-Adipoq-Serpina3c mice compared with AAV8-Adipoq-NC mice (Figure 7E–7F). Our analysis of post-fasting serum lipids revealed decreased TG and NEFA levels in AAV8-Adipoq-Serpina3c mice (Figure 7G). iWAT and eWAT depot weights were lower in AAV8-Adipoq-Serpina3c mice (Supplementary Figure S7A-S7B) compared with AAV8-Adipoq-NC mice. The overall lipid content was lowered in the iWAT and eWAT depots in Serpina3c OV mice (Supplementary Figure S7C-S7D). H&E staining revealed a reduction in the number of hypertrophic adipocytes in iWAT and eWAT depots in AAV8-Adipoq-Serpina3c mice (Figure 7H–7I).

**Figure 8:**
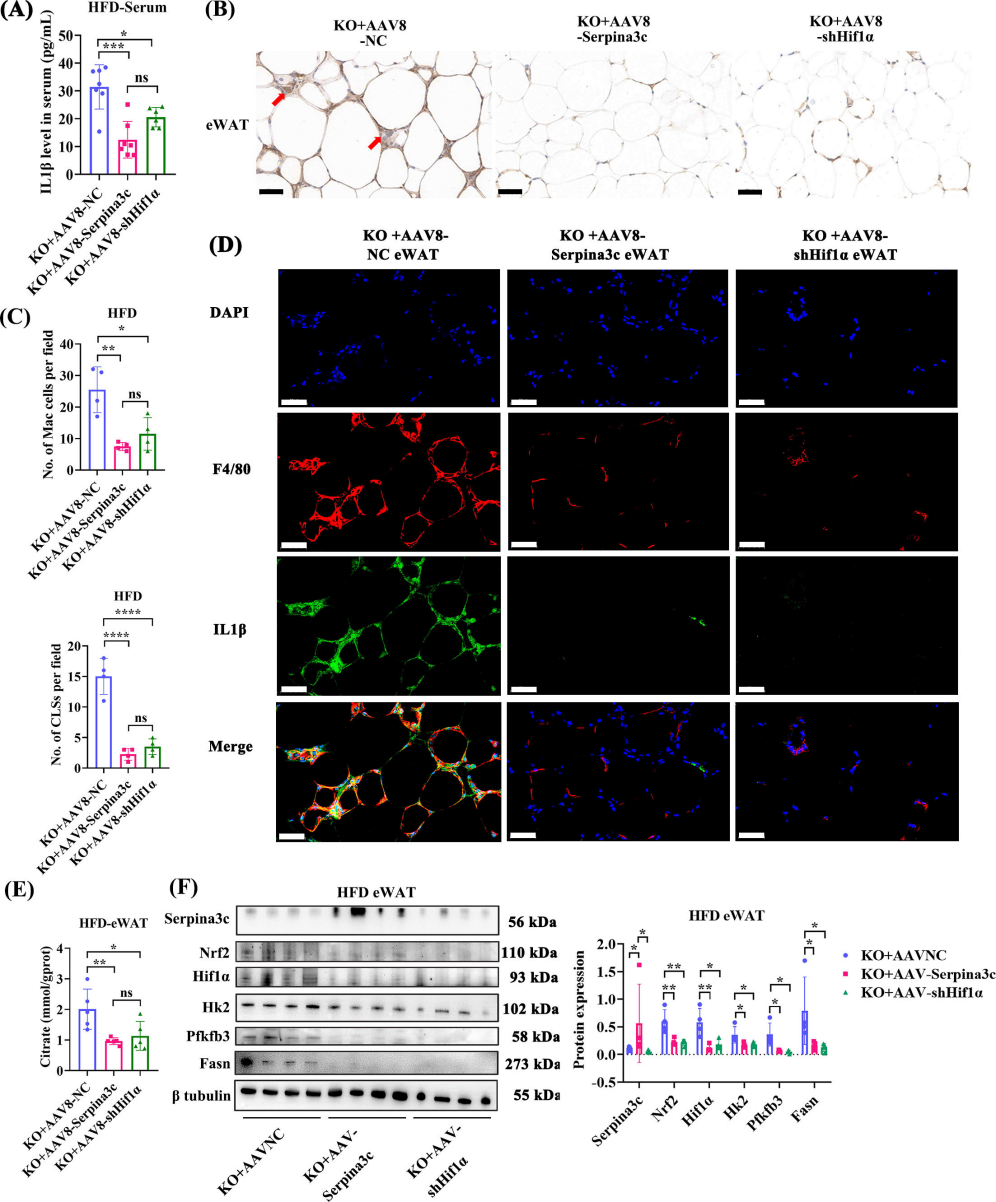
AAV-mediated Serpina3c OV or Hif1α KD improved macrophage-related metaflammation and reduced glycolysis levels. (**A**) Serum IL1β levels in HFD-fed AAV8-Adipoq-NC (*n* = 7), AAV8-Adipoq-Serpina3c (*n* = 7), and AAV8-Adipoq-shHif1α (*n* = 6) mice (*P*<0.05). (**B-C**) Coronal structure and quantification of eWAT from HFD-fed AAV8-Adipoq-NC, AAV8-Adipoq-Serpina3c, and AAV8-Adipoq-shHif1α mice (*n* = 4, *P*<0.05). (**D**) Immunofluorescence staining of F4/80^+^, IL1β, and DAPI in eWAT from HFD-fed AAV8-Adipoq-NC, AAV8-Adipoq-Serpina3c, and AAV8-Adipoq-shHif1α mice (*n* = 3) (bar = 50 μm). (**E**) Citrate content measured in eWAT from HFD-fed AAV8-Adipoq-NC, AAV8-Adipoq-Serpina3c, and AAV8-Adipoq-shHif1α mice (*n* = 5, *P*<0.05). (**F**) Protein expression of Serpina3c, Nrf2, Hif1α, Hk2, Pfkfb3, Fasn, and β-tubulin in eWAT from HFD-fed KO + AAVNC, KO + AAV-Serpina3c and KO + AAV-shHif1α mice (*n* = 4, *P*<0.05). Data are presented as the mean ± SD. ns: not significant, *P < 0.05, **P < 0.01, ***P < 0.001 compared with the control group.

The increased inflammation observed in KO mice could be prevented by AAV8-Adipoq-Serpina3c overexpression (Figure 8A). The immunohistochemical results showed that the number of eWAT CLSs in Serpina3c overexpression group was reduced compared with the control group (Figure 8B–8C). Importantly, the co-localization of F4/80^+^ and IL1β^+^ macrophages was decreased in the eWAT of the AAV8-Adipoq-Serpina3c (Figure 8D). The citrate levels were also decreased in eWAT depots in AAV8-Adipoq-Serpina3c mice (Figure 8E). The expression of Nrf2, Hif1α, Hk2, Pfkfb3, and Fasn was decreased in eWAT from Serpina3c OV mice compared with KO mice (Figure 8F). The liver TG content was decreased in AAV8-Adipoq-Serpina3c mice (Supplementary Figure S7E). H&E staining of liver tissues from AAV8-Adipoq-Serpina3c mice revealed that Serpina3c overexpression also alleviated HFD-induced hepatocyte ballooning and vacuolation (Supplementary Figure S7F). Oil Red O staining demonstrated that lipid droplet deposition was also reduced in the liver (Supplementary Figure S7G). However, there were no noticeable differences in myocardial (Supplementary Figure S7H) and muscle cell morphology in (Supplementary Figure S7I) AAV8-Adipoq-Serpina3c mice and AAV8-Adipoq-NC mice by H&E staining. Oil Red O staining did reveal a reduction in lipid droplet deposition in the hearts (Supplementary Figure S7J) and muscles (Supplementary Figure S7K) of mice in the AAV8-Adipoq-Serpina3c group. In agreement with these observations in AAV8-Adipoq-Serpina3c mice, the lipid metabolism disorders caused by Serpina3c KO were also alleviated in AAV8-mediated Hif1α KD mice on a Serpina3c KO mouse background (Figures 7 and 8, Supplementary Figure S7).

### Serpina3c KO adipocytes promote macrophage-derived inflammation via increased secretion of LD

**Figure 9:**
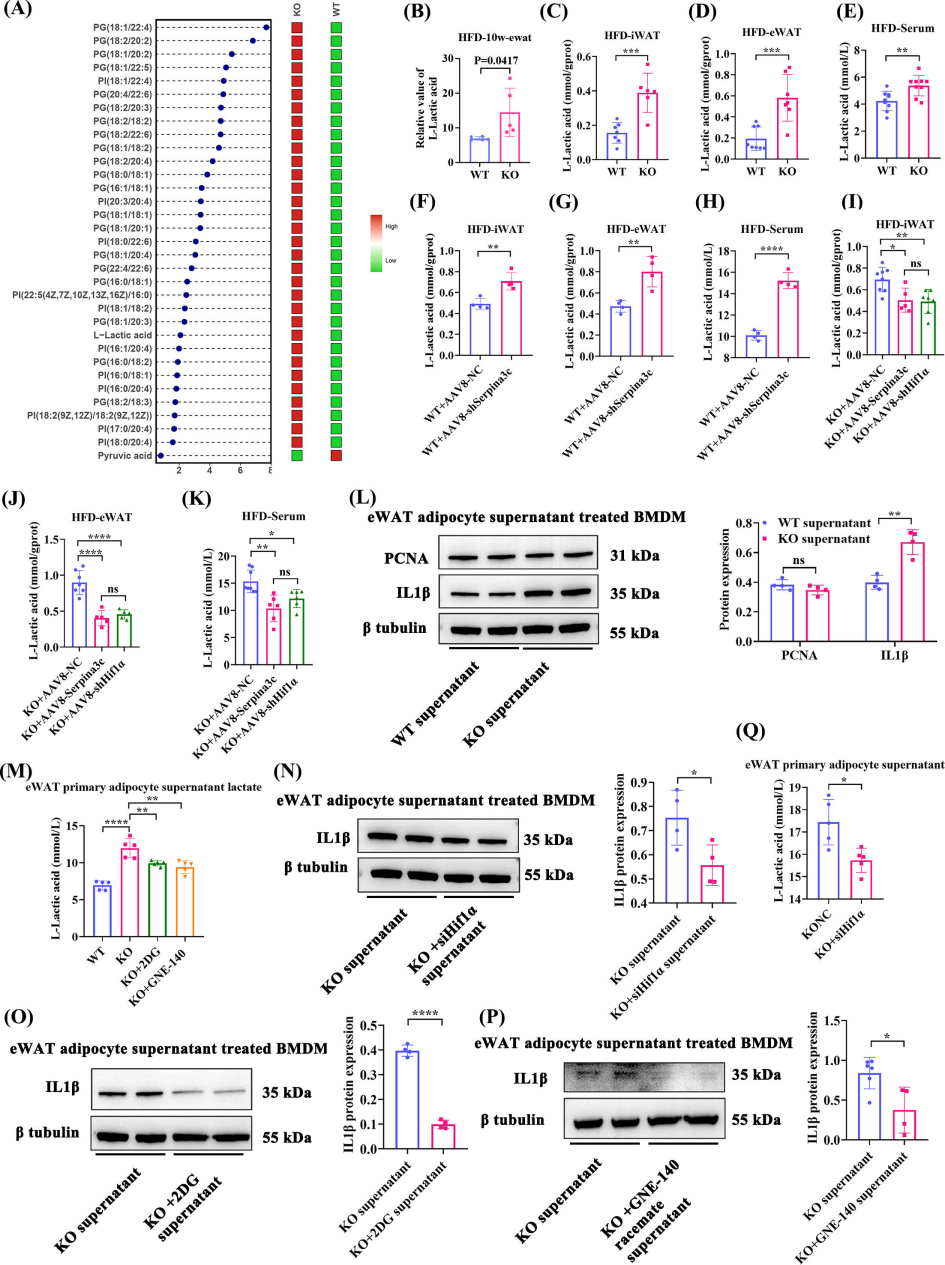
Serpina3c KO in primary adipocytes enhanced macrophage inflammation. (**A**) Representative heatmap of metabolites (*n* = 5). (**B**) LD levels in eWAT from WT and KO mice fed HFD for 10 weeks (*n* = 5; *P*=0.0417, unpaired t-test). (**C-E**) LD levels in iWAT (*P*=0.0006), eWAT (*P*=0.0008), and serum (*P*=0.0049) from WT and KO mice fed HFD for 16 weeks (*n* = 6–9). (**F-H**) iWAT (*P*=0.0123), eWAT (*P*=0.0053) and serum (*P*<0.0001) LD levels in AAV8-Adipoq-NC and AAV8-Adipoq-shSerpina3c mice fed HFD for 16 weeks (*n* = 3–4). (**I-K**) LD levels in iWAT (*P*<0.05), eWAT (*P*<0.001), and serum (*P*<0.05) from AAV8-Adipoq-NC, AAV8-Adipoq-Serpina3c, and AAV8-Adipoq-shHif1α mice fed HFD for 16 weeks (*n* = 5–8). (**L**) Protein expression of PCNA (*P*=0.1875), and IL1β (*P*=0.0013) in WT and KO supernatant-treated BMDMs (*n* = 4). (**M**) LD content in adipocyte culture supernatant in the PA-induced WT, KO, KO+2DG, and KO+GNE140 groups (*n* = 5, *P*<0.001). (**N**) Protein expression of IL1β in KONC and KO+Hif1α KD supernatant-treated BMDMs (*n* = 4, *P*=0.0318). (**O**) Protein expression of IL1β in KO and KO+2DG supernatant-treated BMDMs (*n* = 4, *P*<0.0001). (**P**) Protein expression of IL1β in KO and KO+GNE140 racemate supernatant-treated BMDMs (*n* = 4, *P*=0.0158). (**Q**) LD content in PA-induced KONC supernatant and KO+Hif1α KD supernatant-treated BMDMs (*n* = 5, *P*=0.0106). Data are presented as the mean ± SD. ns: not significant, *P < 0.05, **P < 0.01, ***P < 0.001, ****P < 0.0001 compared with the control group.

In animal experiments, macrophage-associated metaflammation (as represented by F4/80^+^ macrophage infiltration) was increased in Serpina3c KO eWAT depots (Figure 2). However, Serpina3c expression is extremely low in macrophages and high in adipocytes (Supplementary Figure S2G-S2J). We speculated that cross-talk between adipocytes and macrophages plays an important role in inflammation in Serpina3c KO mice after HFD feeding. Metabolomics was performed on eWAT from WT and KO mice fed an HFD for ten weeks to elucidate the underlying processes. The results revealed a tendency for LD levels to be increased in the eWAT depots of KO mice compared with eWAT depots in WT mice (Figure 9A–9B). LD levels in iWAT, eWAT, and serum were significantly increased in KO mice fed an HFD for 16 weeks (Figure 9C–9E) compared with WT mice. The levels of LD were also significantly increased in the iWAT, eWAT, and serum of AAV8-Adipoq-shSerpina3c mice (Figure 9F–9H). This increase in LD levels could be prevented by AAV8-Adipoq-Serpina3c and AAV8-Adipoq-shHif1α treatments (Figure 9I–9K). These findings indicate that LD, a key metabolic byproduct of glycolysis, may play a critical role in regulating macrophage inflammation.

In addition, IL1β protein expression was higher in bone marrow-derived macrophages (BMDMs) treated with conditioned supernatant from Serpina3c KO adipocytes, whereas macrophage proliferation was unchanged (Figure 9L). Consistent with our hypothesis, LD levels were also increased in conditioned supernatant from Serpina3c KO adipocytes (Figure 9M). Conversely, IL1β protein expression was reduced in BMDMs stimulated by conditioned supernatants from Hif1α knockdown adipocytes (Figure 9N), 2-DG-treated adipocytes (Figure 9O), and GNE140 racemate-treated supernatants (Figure 9P). LD levels were decreased in supernatant from Hif1α knockdown adipocytes (Figure 9Q), 2-DG-treated adipocytes (Figure 9M), and GNE140 racemate-treated adipocytes (Figure 9M).

### The plasma expression levels of kallistatin are decreased in obese patients

**Figure 10:**
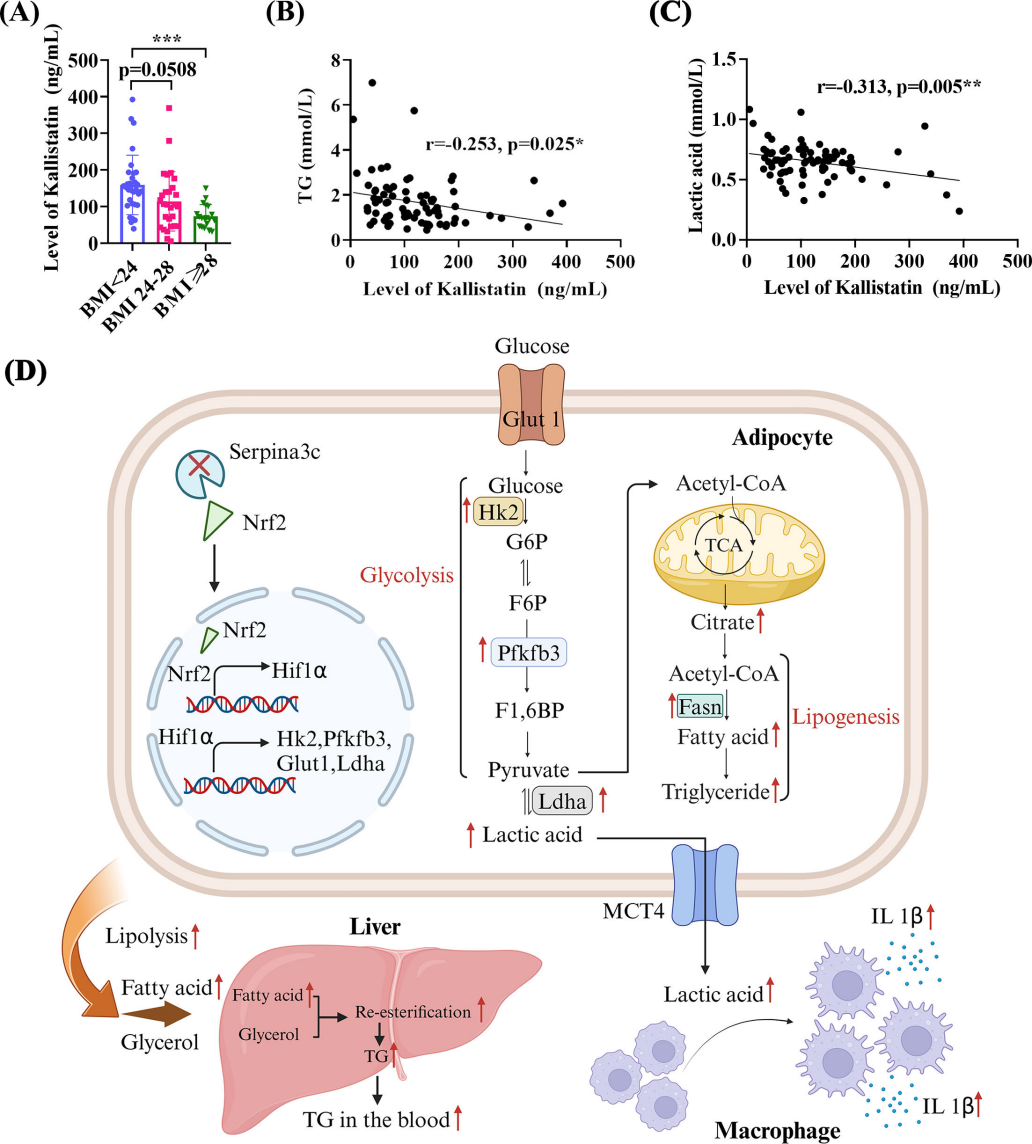
Plasma expression of kallistatin in obese patients. (**A**) Kallistatin expression in the plasma was down-regulated in BMI≥28 group, compared with BMI<24 group (73.38 ± 31.70 vs 159.14 ± 81.27, *P*=0.0003). (**B**) A negative correlation was observed between kallistatin levels and TG, Pearson analysis (*r* = -0.253, *P*=0.025). (**C**) Kallistatin was negatively related to the levels of LD in human plasma (*r* = -0.313, *P*=0.005). (**D**) Schematic diagram of Serpina3c deficiency promoting obesity-related hypertriglyceridemia and inflammation (Created in BioRender. Ji, Z. (2025) https://BioRender.com/q58d916). Under HFD conditions, a reduction in Serpina3c up-regulates Nrf2 expression and subsequently activates the Hif1α pathway, leading to enhanced glycolysis. The products of glycolysis then stimulate fatty acid synthesis, which is stored in the form of triglycerides (TGs). These TGs undergo lipolysis, releasing free fatty acids and glycerol that contribute to the induction of hypertriglyceridemia. Data are presented as mean ± SD. ns: not significant, * *P* < 0.05, ***P < 0.001 compared with control group.

The expression levels of kallistatin (the human homolog of Serpina3c) in plasma were down-regulated in the BMI ≥28 group, compared with the BMI<24 group (Figure 10A; 73.38 ± 31.70 vs 159.14 ± 81.27, *P*=0.0003). A negative correlation was observed between kallistatin levels and TG content (*r* = –0.253, *P*=0.025) (Figure 10B). Kallistatin was negatively related to the levels of LD in human plasma (*r* = –0.313, *P*=0.005; Figure 10C).

## Discussion

Obesity-induced adipose dysfunction is often accompanied by hyperlipidemia [28] and metaflammation [29,30]. Targeting adipose tissue metabolic function is an effective strategy for the future clinical prevention and management of hypertriglyceridemia. In this study, we used *in vivo* and *in vitro* models to demonstrate that adipocyte Serpina3c inhibited Nrf2 expression to reduce Hif1α transcription, thereby reducing glycolysis and DNL, ultimately ameliorating obesity, hypertriglyceridemia, and metaflammation induced by HFD.

First, we found a high expression of Serpina3c in WAT, which could be down-regulated by HFD feeding. Importantly, Serpina3c was highly expressed in adipocytes, but not in undifferentiated SVF preadipocytes and macrophages, suggesting its regulatory role in adipocyte function. Our research revealed that Serpina3c deficiency in adipocytes exacerbated adipocyte dysfunction and obesity-related hypertriglyceridemia after 16 weeks of HFD feeding. We have shown that Serpina3c, which is abundantly expressed in adipocytes, exerts a crucial role in lowering blood lipids and mitigating inflammation by modulating adipocyte function, as evidenced by our findings using AAV8-mediated adipocyte-specific conditional knockout, which led to improved hyperlipidemia and metabolic inflammation.

During the development of obesity, the vascular oxygen supply cannot keep up with the expansion of adipose tissue [31], leading to local hypoxia and Hif1α activation [32]. Our RNA-seq results reveal that the Hif1 and glycolysis pathways were the main pathways up-regulated in PA-treated Serpina3c KD adipocytes. The gene profiles known to be activated by Hif1α include glucose transporters and glycolytic enzymes [33–36]. The *in vivo* and *in vitro* experiments confirmed increases in Hif1α expression and downstream glycolysis-related proteins in the Serpina3c-deleted group compared with the controls. Serpina3c overexpression reduced the expression of Hif1α and glycolysis-related genes. Glycolysis is intimately associated with DNL. Glucose produces pyruvate through glycolysis, which is exported to the mitochondria and utilized as a precursor for citrate, which acts as a direct substrate for DNL. Ultimately, fatty acids are generated by the catalysis of Fasn and stored in the form of TG in lipid droplets of adipocytes [37,38]. Increased DNL and lipolysis in Serpina3c KO adipocytes led to an increase in NEFA release from adipose tissues, further promoting TG synthesis in the liver and severe hypertriglyceridemia [39,40]. These findings demonstrate that the glycolytic pathway and the transcription factor Hif1α are important factors contributing to the accumulation of fat droplets in adipocytes following Serpina3c deletion. Based on these observations, we propose that elevated Hif1α levels in the absence of Serpina3c promote adipocyte hypertrophy, further exacerbating adipose tissue hypoxia. In turn, this adipose tissue hypoxia further promotes increases in Hif1α. Serpina3c overexpression, Hif1α knockdown, and glycolysis inhibition, along with alleviated adipocyte hypertrophy and decreased lipid content, collectively indicate that Serpina3c improves hypertriglyceridemia via the Hif1α/glycolysis pathway.

Additionally, we observed that Serpina3c deficiency led to an increase in Nrf2 localization to the nucleus. NRF2 has been reported to bind to the HIF1α promoter, transcriptionally regulating HIF1α mRNA levels [41]. Nrf2 is a member of the (basic leucine zipper) bZIP transcription factor Cap‘n’Collar (CNC) subfamily and is capable of inducing the expression of a series of antioxidant proteins [42,43]. Previous research on Serpina3c has mainly focused on its function as an exocrine protein and its binding to membrane receptors in mature adipocytes. For instance, kallistatin binds with KLF4 to regulate endothelial nitric oxide synthase expression in endothelial cells [20], and Serpina3c can bind with Nr4a1 to regulate the acetylation of fibroblasts [21]. In the present study, we discovered that Serpina3c, which is highly expressed in adipocytes, binds and inhibits the expression of Nrf2. In previous research, Nrf2 promoted lipid accumulation in adipocytes by activating SREBP-1-mediated lipogenesis [44] and also maintained the shape of lipid droplets and increased TG storage [45]. In our study, we found Serpina3c KO promoted increased transcription of Hif1α and affected downstream processes such as glycolysis and lipogenesis via Nrf2 [46].

Metaflammation is known to be related to dyslipidemia and obesity [47]. The hallmark of adipose tissue metaflammation is the accumulation and polarization of immune cells, along with an increased release of pro-inflammatory cytokines [48]. During obesity, adipose tissue macrophages account for 40% of the total number of adipose tissue cells, compared with 10% under normal conditions [49]. Serpina3c KO mice demonstrated increased macrophage inflammation in adipose tissue, which could be rescued by Serpina3c overexpression or Hif1α knockdown. Despite the lack of Serpina3c expression in macrophages, an increase in the number of pro-inflammatory macrophages in WAT depots was observed in Serpina3c KO mice. Our metabolomics results suggest that LD serves as a link between adipocytes and macrophages and show a slightly increasing trend in KO mice after 10 weeks of HFD feeding, reaching a significant difference by 16 weeks of feeding. In *in vitro* experiments, KO supernatant with lactate inhibitor could reduce macrophage inflammation. Thus, the increase in lactate was a consequence of obesity and a contributor to increased macrophage inflammation. Indeed, in obese adipose tissue, glycolysis and Hif1α activation in eWAT cause an increase in LD synthesis by adipocytes. Importantly, this increase is not generated by SVF or myeloid cells [50]. The large amount of LD produced by adipocytes in the obese state promotes macrophage inflammation and contributes to the formation of a pro-inflammatory micro-environment in eWAT [51]. Therefore, increased LD secretion by Serpina3c KO adipocytes can affect the pro-inflammatory phenotype of macrophages. We also demonstrated that kallistatin levels in plasma from patients decreased with increasing BMI, and were negatively correlated to TG and LD. Thus, Serpina3c/kallistatin is a promising target for the treatment of obesity-related hypertriglyceridemia and metaflammation.

However, there are still some limitations in our study. First, we were unable to obtain human adipose tissue samples for validation. Second, we have not yet developed a drug delivery strategy that can specifically target kallistatin/Serpina3c within adipocytes, with the aim of providing new options for translational research in obesity treatment.

## Conclusion

In conclusion, Serpina3c regulates adipocyte function to improve hypertriglyceridemia and macrophage inflammation via the Hif1α/glycolysis pathway. Serpina3c is a promising target for the treatment of obesity-related hypertriglyceridemia and metaflammation.

## Clinical perspectives

- Obesity-induced adipose dysfunction is often accompanied by hyperlipidemia and metaflammation. Targeting adipose tissue metabolic function is an effective strategy for the future clinical prevention and management of hypertriglyceridemia.
- Serpina3c inhibits the Hif1α-glycolysis pathway and reduces de novo lipogenesis and lactic acid secretion in adipocytes by binding to Nrf2, ultimately ameliorating obesity, hypertriglyceridemia, and metaflammation induced by HFD. Serpina3c knockout aggravates these phenotypes in HFD mice. The plasma expression levels of kallistatin (the human homolog of Serpina3c) are decreased in obese patients and are negatively related to triglyceride and lactic acid content in human plasma.
- Serpina3c/kallistatin is a promising target for the treatment of obesity-related hypertriglyceridemia and metaflammation.

## Supplementary Material

Figure S1

Online supplementary table 1

## Data Availability

All data generated or analyzed during this study are included in this published article [and its supplementary information files]. The raw sequence data reported in the present paper have been deposited in the Genome Sequence Archive (Genomics, Proteomics & Bioinformatics 2021) at the National Genomics Data Center (Nucleic Acids Res 2022), China National Center for Bioinformation / Beijing Institute of Genomics, Chinese Academy of Sciences (Accession: PRJCA029932; CRA018857; CRA018906) that are publicly accessible at https://ngdc.cncb.ac.cn/gsa.

## Open Access

This article has been published open access under our Subscribe to Open programme, made possible through the support of our subscribing institutions, learn more here: https://portlandpress.com/pages/open_access_options_and_prices#conditional

## Abbreviations

AAV8: AAV of serotype 8
ALT: alanine aminotransferase
AST: aspartate aminotransferase
ATGL: adipose triglyceride lipase
ApoA4: Apolipoprotein A-IV
BAT: brown adipose tissue
BCA: bicinchoninic acid
BM: bone marrow
BMDM: bone marrow-derived macrophage
BMI: body mass index
CAD: coronary artery diseases
CLS: crown-like structure
CNC: Cap’-n’-Collar
CO-IP: co-immunoprecipitation
DAPI: 4,6-diamino-2-phenyl indole
DMEM: Dulbecco’s modified Eagle medium
DNL: *de novo* lipogenesis
ECAR: extracellular acidification rate
EDTA: ethylenediaminetetraacetic acid‌
Fasn: fatty acid synthase
GSEA: gene set enrichment analysis
Glut-1: glucose transporter-1
HDL-C: high-density lipoprotein cholesterol
H&E: hematoxylin-eosin
HFD: high-fat diet
Hif1α: hypoxia-inducible factor 1 α
Hk2: hexokinase 2
IBMX: isobutylmethylxanthine
KD: knockdown
KEGG: Kyoto Encyclopedia of Genes and Genomes
LC-MS: liquid chromatography mass spectrometry
LD: lactic acid
LDL-C: low-density lipoprotein cholesterol
Ldha: lactate dehydrogenase A
MRI: micro-magnetic resonance imaging
NC: normal control
ND: normal diet
NEFA: non-esterified fatty acid
Nrf2: nuclear factor erythroid 2-related factor 2
OV: overexpression
PA: palmitic acid
PCR: polymerase chain reaction
PER: proton efflux rate
PVDF: ‌polyvinylidene fluoride‌
Pfkfb3: 6-phosphofructo-2-kinase/ fructose-2,6-biphosphatase 3
RBC: red blood cells
SVF: stromal vascular fraction
Serpin: serine protease inhibitor
TC: total cholesterol
TG: triglyceride
VAT: visceral adipose tissue
WAT: white adipose tissue
WAT: white adipose tissue
WT: wild type
bZIP: basic leucine zipper
eWAT: epididymal white adipose tissue
iWAT: inguinal WAT
qRT-PCR: quantitative real-time PCR
-seq: RNA sequencing
