# Supplementary material for: Serpina3c deficiency promotes obesity-related hypertriglyceridemia and inflammation through activation of the Hif1α-glycolysis axis in adipose tissue
Source: Clin Sci (Lond). 2025 Aug 28;139(16):897–918. doi: 10.1042/CS20242610 (PMC12493168; doi:10.1042/CS20242610)
Supplement: Figure S1 [file cs-139-16-CS20242610-s001.pdf]

Supplementary Figures

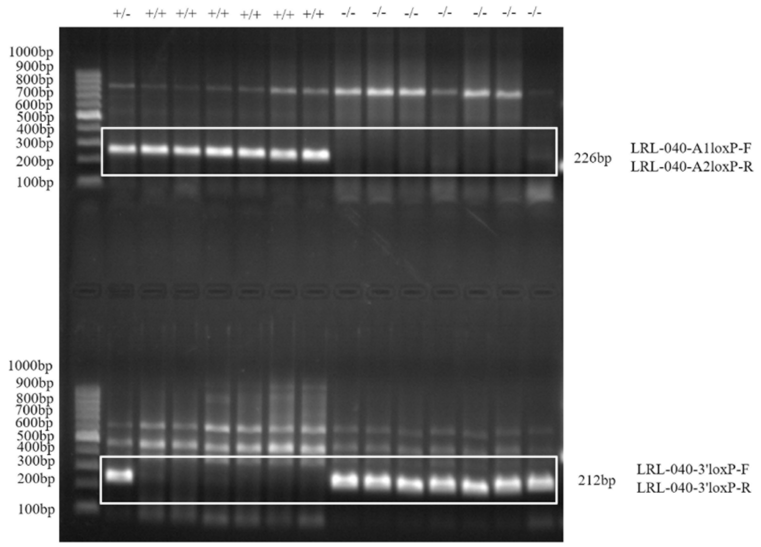

Supplementary Figure 1. Generation and identification of Serpina3c gene knockout (KO) mice.

Genotyping information of WT, heterozygous mice, and Serpina3c KO mice in the C57BL/6 background. Mice used in this study were mated by the heterozygous mice.

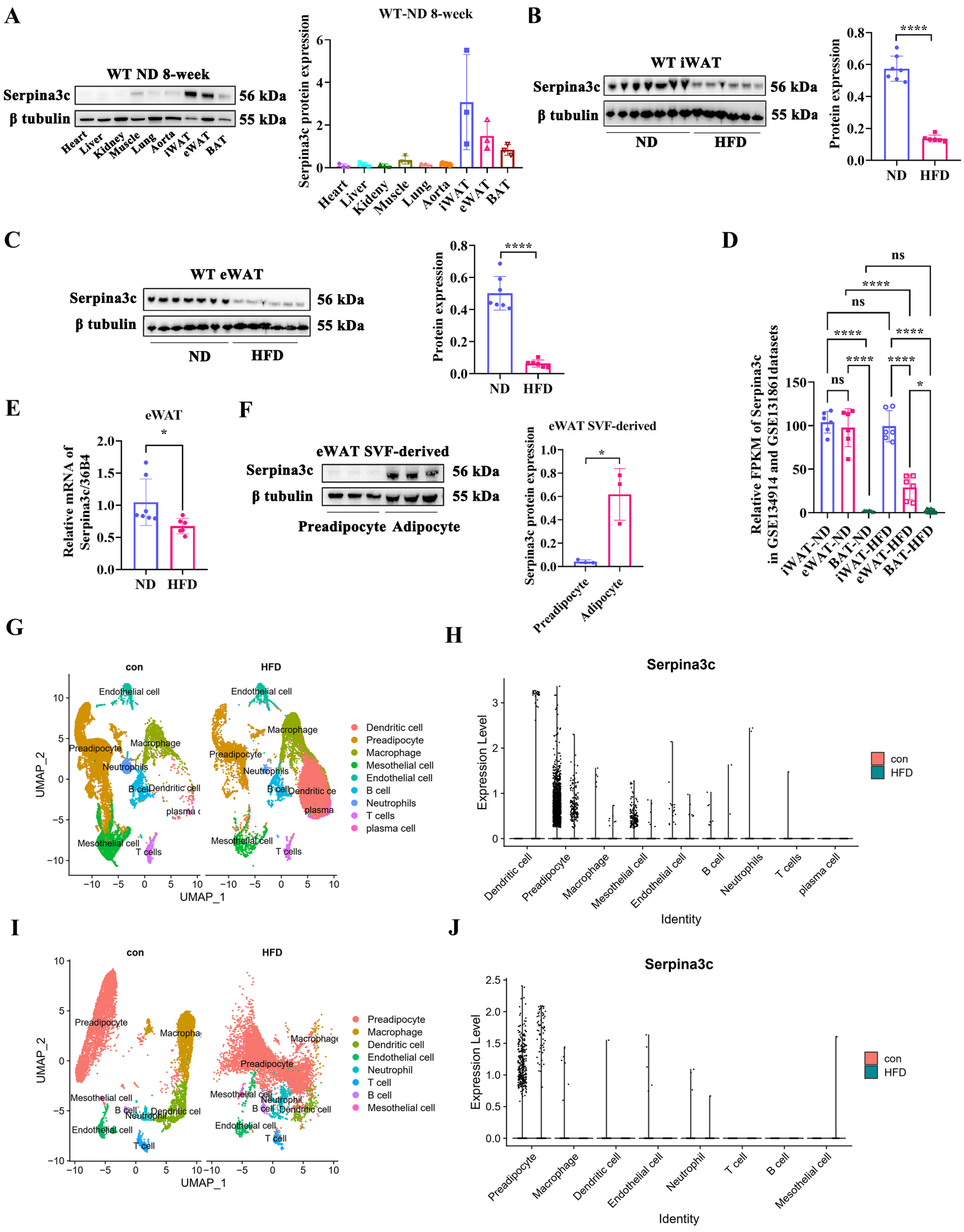

Supplementary Figure 2. The expression of Serpina3c in adipose tissue, SVF and adipocytes.

A The protein expression of Serpina3c in male WT mice ($n = 3$). **B** Serpina3c protein expression in iWAT in the ND ($n = 7$) and HFD ($n = 6$) groups ($p < 0.0001$, unpaired t-test). **C** Serpina3c protein expression in eWAT in the ND ($n = 7$) and HFD ($n = 6$) groups

($p < 0.0001$, unpaired t-test). **D** Analysis of RNA-sequencing datasets (GSE134914 and GSE131861) showing Serpina3c expression in iWAT and eWAT in ND and HFD mice. **E** Serpina3c mRNA expression in eWAT in the ND ($n = 7$) and HFD ($n = 6$) groups ($p = 0.036$, unpaired t-test). **F** The protein expression of Serpina3c in WT eWAT SVF-derived preadipocytes and adipocytes ($n = 3$; $p = 0.0108$, unpaired t test). **G** Analysis of single-cell sequencing datasets (GSE161872) showing different cell clusters in eWAT in ND and HFD mice. **H** Analysis of single-cell sequencing datasets (GSE161872) showing Serpina3c expression in different cell clusters from eWAT of ND and HFD mice. **I** Analysis of single-cell sequencing datasets (GSE237143) showing different cell clusters in eWAT in ND and HFD mice. **J** Analysis of single-cell sequencing datasets (GSE237143) showing Serpina3c expression in different cell clusters from eWAT of ND and HFD mice. Values are presented as the mean ± SD. ns: not significant, *$p < 0.05$, ****$p < 0.0001$ compared to the control group.

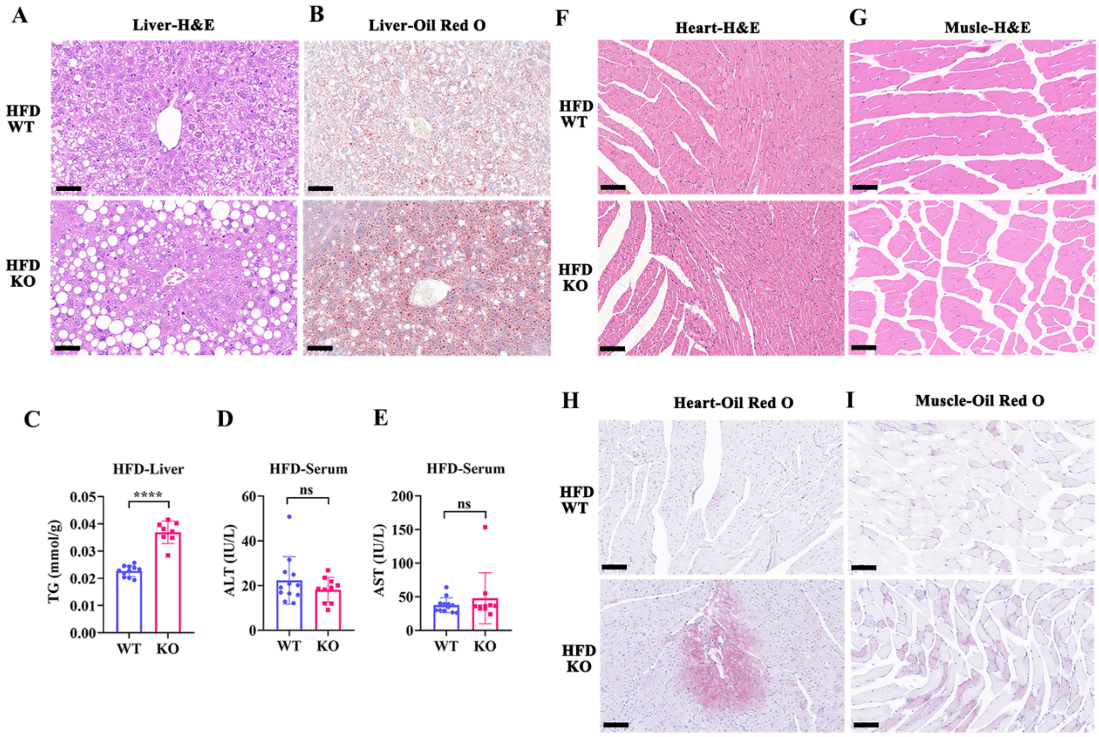

Supplementary Figure 3. Serpina3c deletion aggravated HFD-induced ectopic fat storage.

A H&E staining of liver from HFD-fed WT and KO mice (n = 5; left: bar = 200 μm, right: enlarged 4 times). **B** Oil red O staining of liver from HFD-fed WT and KO mice (n = 5; left: bar = 200 μm, right: enlarged 4 times). **C** TG content in livers from HFD-fed WT (n = 11) and KO (n = 8) mice ($p < 0.0001$, unpaired t-test). **D** Serum ALT levels in HFD-fed WT (n = 12) and KO (n = 10) mice ($p = 0.2748$, unpaired t-test). **E** Serum AST levels in HFD-fed WT (n = 12) and KO (n = 10) mice ($p = 0.3738$, unpaired t-test). **F** H&E staining of hearts from HFD-fed WT and KO mice (n = 3; bar = 100 μm). **G** H&E staining of muscle from HFD-fed WT and KO mice (n = 3; bar = 100 μm). **H** Oil red O staining of hearts from HFD-fed WT and KO mice (n = 3; bar = 100 μm). **I** Oil red O staining of muscle from HFD-fed WT and KO mice (n = 4; bar = 100 μm).

Data are shown as the mean ± SD. ns: not significant, *p < 0.05, ****p < 0.0001 compared to the control group.

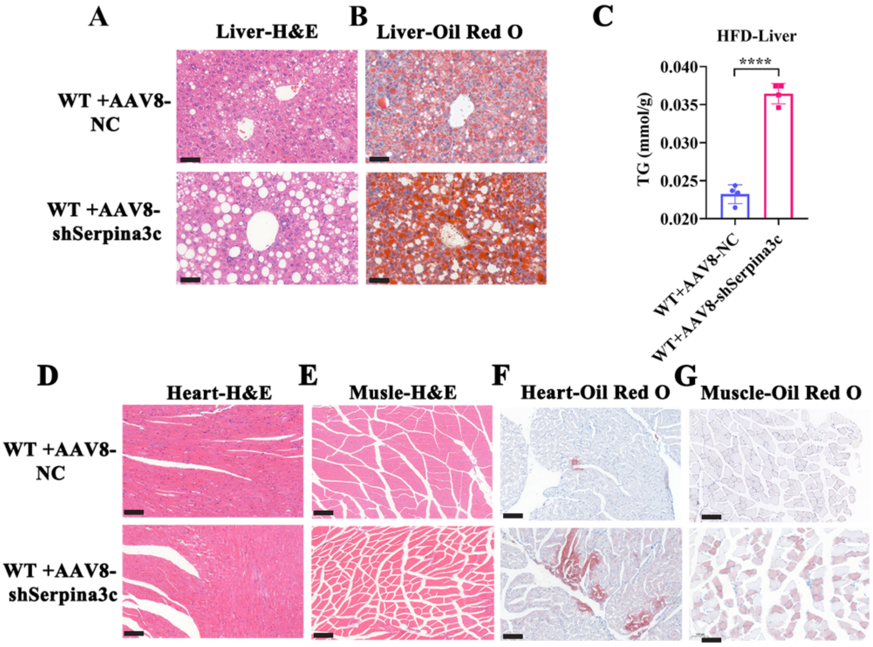

Supplementary Figure 4. AAV8 mediated adipocyte-specific KD of Serpina3c increased HFD-induced ectopic fat storage.

A H&E staining of liver from HFD-fed AAV8-Adipoq-shSerpina3c mice showed more severe steatosis (n = 5) than AAV8-Adipoq-NC (n = 4) mice (Left: bar = 200 μm, right: enlarged 4 times). **B** Oil Red O staining further confirmed higher steatosis in the liver of HFD-fed AAV8-Adipoq-shSerpina3c mice (n = 5) compared to AAV8-Adipoq-NC (n = 4) mice (Left: bar = 200 μm, right: enlarged 4 times). **C** TG content was higher in liver from HFD-fed AAV8-Adipoq-shSerpina3c mice than AAV8-Adipoq-NC mice (n

= 4; $p < 0.0001$, unpaired t test). **D** H&E staining demonstrated no changes in the morphology of cardiomyocytes between the HFD-fed AAV8-Adipoq-NC and AAV8-Adipoq-shSerpina3c mice (n = 4, bar = 100 μm). **E** H&E staining demonstrated no changes in the morphology of skeletal muscle cells between the HFD-fed AAV8-Adipoq-NC (n = 4) and AAV8-Adipoq-shSerpina3c (n = 5) mice (bar = 100 μm). **F** Oil Red O staining further confirmed higher steatosis in the heart of AAV8-Adipoq-shSerpina3c mice compared to AAV8-Adipoq-NC mice (n = 4, bar = 100 μm). **G** Oil Red O staining further confirmed higher steatosis in the muscle of HFD-fed AAV8-Adipoq-shSerpina3c mice compared to AAV8-Adipoq-NC mice (n = 4, bar = 100 μm). In summary, these results indicated that Serpina3c deficiency in adipose tissue exacerbated deterioration of ectopic fat storage. Data are presented as mean ± SD. ****$p < 0.0001$ compared to control group.

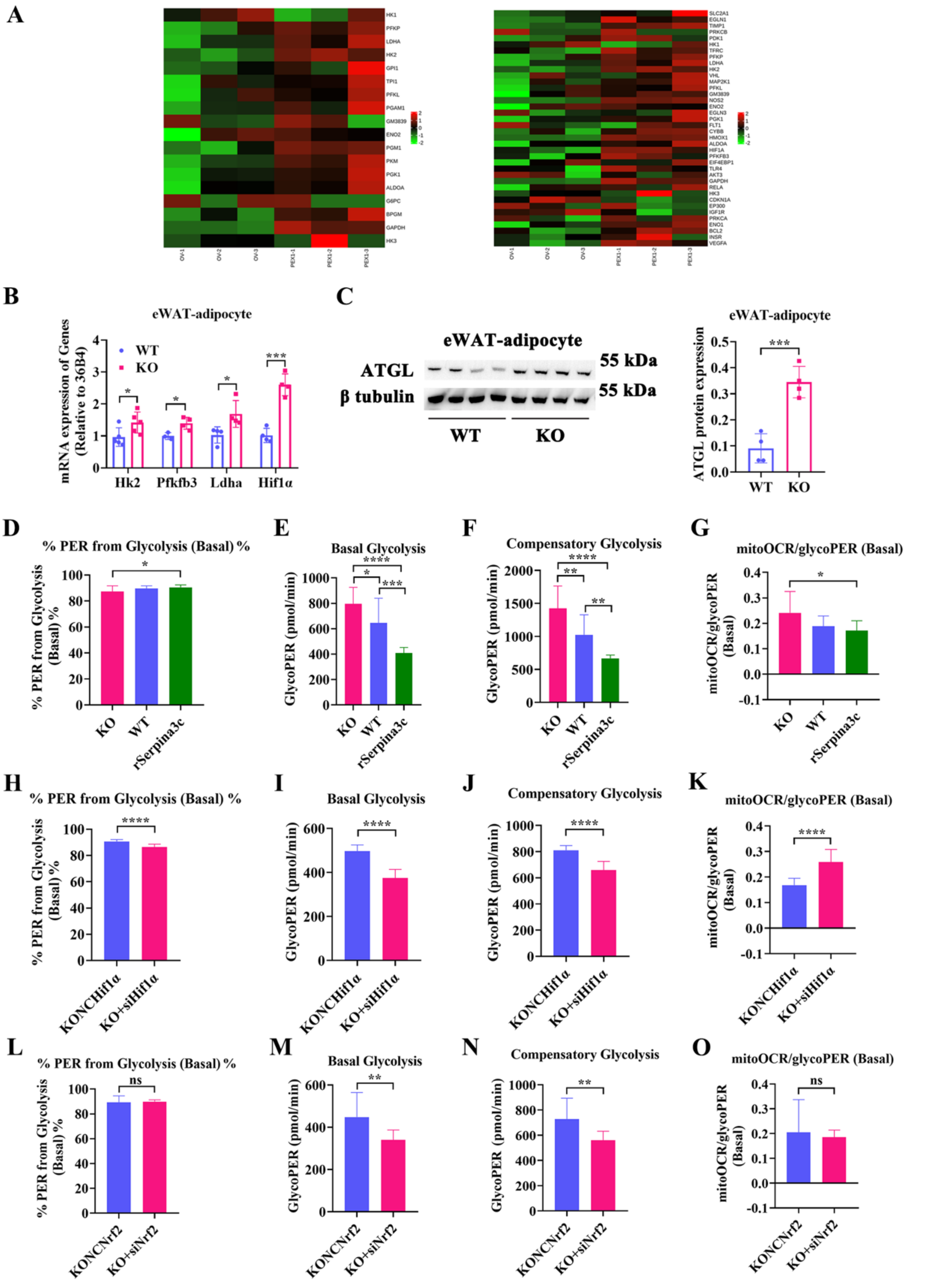

Supplementary Figure 5. ECAR levels in adipocytes.

A Heat map of RNA-seq in PA-induced Serpina3c OV 3T3-L1 adipocytes. **B** mRNA expression of Hk2 (p = 0.0485), Pfkfb3 (p = 0.0214), Ldha (p = 0.0363), and Hif1α (p

= 0.0002) in PA-induced WT and Serpina3c KO primary adipocytes (n = 3–5). **C** The ATGL protein expression in PA-treated WT and KO eWAT SVF-derived adipocytes (n = 4, p = 0.0008). **D** The %PER from glycolysis of WT, KO and KO treated with recombinant protein Serpina3c groups (n = 4; $p < 0.05$, one-way ANOVA). **E-G** Quantitative data of basal glycolysis, compensatory glycolysis and basal mitoOCR/glycoPER ratio in WT, KO and KO treated with recombinant protein Serpina3c groups, (n = 4; $p < 0.05$, one-way ANOVA). **H** The %PER from glycolysis of NC and siHif1α groups (n = 4; p <0.0001, unpaired t test). **I-K** Quantitative data of basal glycolysis, compensatory glycolysis and basal mitoOCR/ glycoPER ratio in NC and siHif1α groups (n = 4; $p < 0.0001$, unpaired t test). **L** The %PER from glycolysis of NC and siNrf2 groups (n = 4; $p > 0.05$, unpaired t test). **M-O** Quantitative data of basal glycolysis ($p < 0.01$), compensatory glycolysis ($p < 0.01$) and basal mitoOCR/glycoPER ratio ($p > 0.05$) in NC and siNrf2 groups (n = 4, unpaired t test). Data are presented as mean ± SD. *p < 0.05, **p < 0.01, ****p < 0.0001 compared to control group.

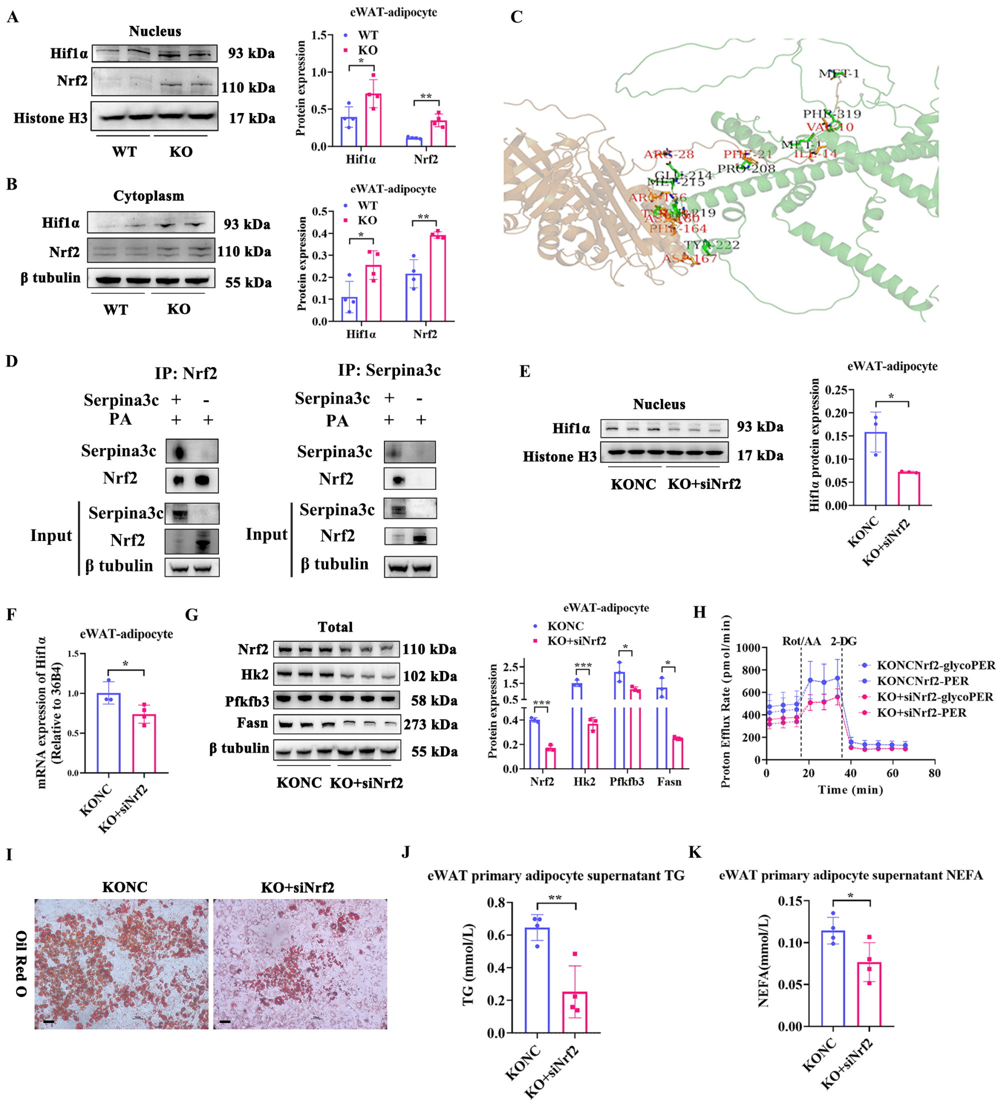

Supplementary Figure 6. Serpina3c KO facilitated the expression of Nrf2, the upstream transcriptional regulator of Hif1α.

A Nuclear protein expression of Hif1α (p = 0.0355), Nrf2 (p = 0.0016), and histone H3 in WT and KO adipocytes (n = 4; unpaired t-test). **B** Cytoplasmic protein expression of Hif1α (p = 0.0239), Nrf2 (p = 0.0017), and β tubulin in WT and KO adipocytes (n = 4; unpaired t-test). **C** Schematic diagram of binding interface interaction residues. Nrf2-Q60795-model by green cartoon model display, Serpina3c-P29621-model was shown in orange. Residues with interfacial interaction energy lower than -8kcal/mol were used

as s tick model showed that residues from Nrf2-Q60795-model were marked with black residue name and serial number, and residues from Serpina3c-P29621-model were marked with red residue name and serial number. The contact residues were defined by the proximity of receptor residues to ligand residues within a distance of 4 Å. The energy contribution of the receptor-ligand contact residue pairs was then calculated based on this definition. The energy contribution of the interaction between the contact residues on Nrf2, which includes PHE319, MET1, PRO208, GLU214, MET215, TYR219, TYR222, and TYR219, and the contact residues on Serpina3c, which includes VAL10, ILE14, PHE21, ARG28, ARG156, PHE164, ASP167, ASP180, and TYR181, was found to be lower than -8.0 kcal/mol. **D** Co-IP assay using Nrf2 antibody and Serpina3c antibody in PA treated WT and Serpina3c KO adipocytes derived from SVF (n = 3). **E** Nuclear protein expression of Hif1α and histone H3 in KONC and KO+Nrf2 KD adipocytes (n = 3; p = 0.0259, unpaired t-test). **F** mRNA expression of Hif1α in PA-induced KONC and KO+Nrf2 KD adipocytes (n = 4; p = 0.0382, unpaired t-test). **G** Total protein expression of Nrf2 (p = 0.0002), Hk2 (p = 0.0006), Pfkfb3 (p = 0.0399), Fasn (p = 0.0456), and β tubulin in KONC and KO+Nrf2 KD adipocytes (n = 3; unpaired t-test). **H** Seahorse glycolysis stress test with the sequential addition of Rot/AA and 2-DG in PA-induced KONC and KO+siNrf2 adipocyte-derived from SVF (n = 4). **I** Oil Red O staining in PA-induced KONC and KO+siNrf2 adipocytes (n = 4) (bar = 50 μm). **J** TG content in adipocyte culture supernatant in the PA-induced KONC and KO+Nrf2 KD groups (n = 4; p = 0.0045, unpaired t-test). **K** NEFA content in adipocyte culture supernatant in the PA-induced KONC and KO+Nrf2 KD groups (n =

4; $p = 0.0369$, unpaired t-test). Data are presented as the mean ± SD. *$p < 0.05$, **$p < 0.01$, ***$p < 0.001$ compared to the control group.

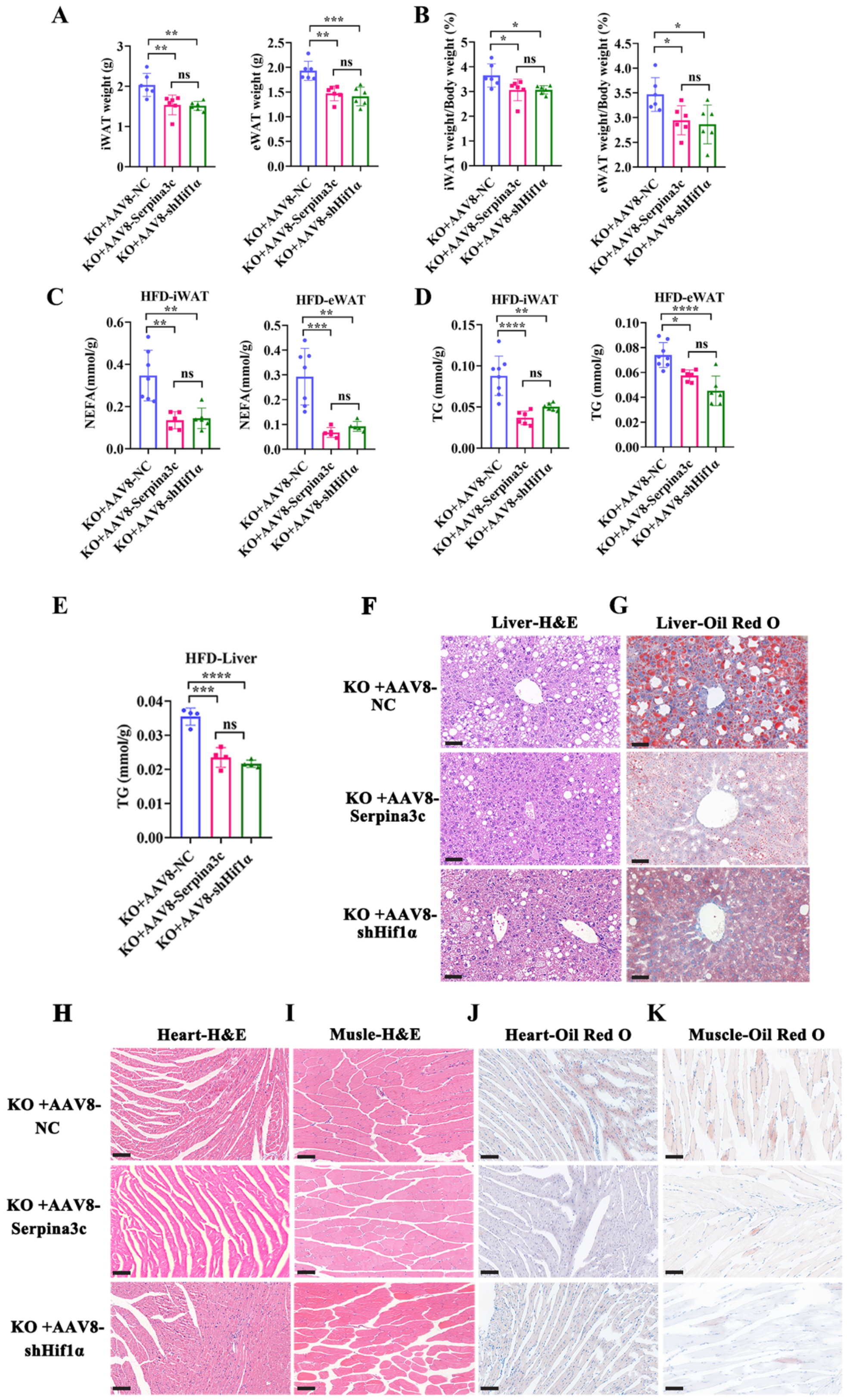

Supplementary Figure 7. AAV8-mediated adipose-specific overexpression of Serpina3c or knockdown of Hif1α improved HFD-induced ectopic fat storage.

A iWAT ($n = 6$, $p < 0.01$) and eWAT ($n = 6$, $p < 0.001$) weight in three groups. **B** The proportion of iWAT weight to body weight ($n = 6$, $p < 0.05$) and eWAT weight to body weight ($n = 6$, $p < 0.05$) in HFD-fed AAV8-Adipoq-NC, AAV8-Adipoq-Serpina3c, and AAV8-Adipoq-shHif1α mice. **C** NEFA levels measured in iWAT ($n = 5$-7, $p < 0.01$) and eWAT ($n = 5$-7, $p < 0.001$) from three groups. **D** TG in iWAT ($n = 6$-8, $p < 0.001$) and eWAT ($n = 6$-8, $p < 0.05$) from three groups. **E** TG content in livers from HFD-fed AAV8-Adipoq-NC, AAV8-Adipoq-Serpina3c, and AAV8-Adipoq-shHif1α mice ($n = 4$; $p < 0.05$, one-way ANOVA and Tukey's test). **F** H&E staining of liver from HFD-fed AAV8-Adipoq-NC, AAV8-Adipoq-Serpina3c, and AAV8-Adipoq-shHif1α mice ($n = 5$-6; left: bar = 200 μm, right: enlarged 4 times). **G** Oil Red O staining of liver from HFD-fed AAV8-Adipoq-NC, AAV8-Adipoq-Serpina3c, and AAV8-Adipoq-shHif1α mice ($n = 5$-6; left: bar = 200 μm, right: enlarged 4 times). **H** H&E staining of hearts from HFD-fed AAV8-Adipoq-NC, AAV8-Adipoq-Serpina3c, and AAV8-Adipoq-shHif1α mice ($n = 5$-6; bar = 100 μm). **I** H&E staining of muscle from HFD-fed AAV8-Adipoq-NC, AAV8-Adipoq-Serpina3c, and AAV8-Adipoq-shHif1α mice ($n = 4$-6; bar = 100 μm). **J** Oil Red O staining of hearts from HFD-fed AAV8-Adipoq-NC, AAV8-Adipoq-Serpina3c, and AAV8-Adipoq-shHif1α mice ($n = 4$-5; bar = 100 μm). **K** Oil Red O staining of muscle from HFD-fed AAV8-Adipoq-NC, AAV8-Adipoq-Serpina3c, and AAV8-Adipoq-shHif1αmice ($n = 4$-5; bar = 100 μm). Data are presented as the mean ± SD. ns: not significant, ***$p < 0.001$, ****$p < 0.0001$ compared to the control group.
