# Supplementary material for: Serpina3c deficiency promotes obesity-related hypertriglyceridemia and inflammation through activation of the Hif1α-glycolysis axis in adipose tissue
Source: Clin Sci (Lond). 2025 Aug 28;139(16):897–918. doi: 10.1042/CS20242610 (PMC12493168; doi:10.1042/CS20242610)
Supplement: Online supplementary table 1 [file cs-139-16-CS20242610-s002.pdf]

Supplementary Tables

Supplementary Table 1. List of primers used in the study.

Mouse-36B4-F	ACTGCTGAACATGCTGAACATCTC
Mouse-36B4-R	AGGGTTATAAATGCTGCCGTTGTC
Mouse-Serpina3c-F	ATCTCAGACCTGGATACGGATACAC
Mouse-Serpina3c-R	CACCTTCACAGACCTCTTCACATC
Mouse-Glut1-F	AGAAGAGGGTCGGCAGATGATG
Mouse-Glut1-R	TTGAGTAGTAGAACACAGCATTGATACC
Mouse-Hif1α-F	GAATGAAGTGCACCCTAACAAG
Mouse-Hif1α-R	GAGGAATGGGTTCACAAATCAG
Mouse-Hk2-F	CTACATGGAGGAGATGCGTAAT
Mouse-Hk2-R	GCTTTGTGAAATCGATCAGGAT
Mouse-Pfkfb3-F	TTGTCCAGCAGAGGCAAGAAGTTC
Mouse-Pfkfb3-R	CACACACGGAGGTCCTTCAGATTC
LRL-040-A1loxP-F	CTGCTCATGATGTAGGAGATCTTAGC
LRL-040-A2loxP-R	TCCAGTCATGGTTTGGATAGTGG
LRL-040-3'loxP-F	CTGCTCATGATGTAGGAGATCTTAGC
LRL-040–3'loxP-R	TCTGTAGGCCTGGATCCACCTAAT
Hif1α siRNA-sense	sense 5′-GAGCUUUGGAUCAAGUUAATT-3′
Hif1α siRNA-anti-sense	antisense 5′-UUAACUUGAUCCAAAGCUCTT-3′

Nrf2 siRNA-sense	sense 5´-CAGGAGAAUUCCUCCCAAUTT-3´
Nrf2 siRNA-anti-sense	antisense 5´-AUUGGGAGGAAUUCUCCUGTT-3´

Supplementary Table 2. List of antibodies used in the study.

**Antibody**	**Catalog**	**Company**
Serpina3c	50375-RP01	SinoBiological
β tubulin	T0023	Affinity
Hif1α	BF8002	Affinity
Hk2	Ab209847	Abcam
Pfkfb3	Ab181861	Abcam
Fasn	Ab128870	Abcam
Nrf2	66504-1-Ig	Proteintech
Histone H3	17168-1-AP	Proteintech
PCNA	60097-1-Ig	Proteintech
IL1β	D6D6T	Cell Signaling Technology
F4/80	GB113373	Servicebio
Mac2	GB12940	Servicebio
IL1β	ab283818	Abcam
Adiponectin	66239-1-Ig	Proteintech
Cy3 Labeled Goat Anti-Rabbit IgG	GB21303	Servicebio
Alexa Fluor 488 labeled Goat Anti-	GB25301	Servicebio

mouse IgG		
Polymer-HRP general-purpose rat/rabbit secondary antibody kit	AFIHC001	AiFang biological
Adipose Triglyceride Lipase	ab109251	Abcam

Supplementary Table 3. Basic characteristics of patients with or without obesity.

Characteristics	BMI < 24 (n=31)	BMI 24-28 (n=28)	BMI ≥ 28 (n=19)	P value
**Sex (female)**	15 (48.39%)	16 (57.14%)	9 (47.37%)	0.739
**Smoking**	8 (25.81%)	6 (21.43%)	5 (26.32%)	0.902
**Diabetes**	5 (16.13%)	6 (21.43%)	6 (31.58%)	0.438
**Hypertension**	15 (48.39%)	17 (60.71%)	14 (73.68%)	0.205
**BMI**	22.28 ± 1.44	25.86 ± 1.34	29.75 ± 2.10	p<0.001
**Age (years)**	63.42 ± 9.84	61.00 ± 7.94	60.47 ± 11.08	0.486
**SBP (mmHg)**	133.32 ± 19.58	132.14 ± 18.82	127.53 ± 18.00	0.563
**WBC (10^9/L)**	6.04 ± 1.90	6.66 ± 1.68	6.45 ± 1.88	0.436
**Hb (g/L)**	135.81 ± 13.92	138.68 ± 11.04	137.53 ± 14.70	0.702
**ALT (U/L)**	20.94 ± 9.15	27.57 ± 14.99	26.89 ± 15.35	0.115
**AST (U/L)**	21.61 ± 5.61	23.96 ± 8.08	23.26 ± 9.45	0.479
**BUN (mmol/L)**	5.19 ± 1.24	5.56 ± 1.295	6.12 ± 2.27	0.133
**UA (μmol/L)**	308.23 ± 93.56	328.14 ± 86.70	348.89 ± 87.06	0.296
**Creatinine (μmol/L)**	69.03 ± 18.67	67 ± 21.53	70.21 ± 21.98	0.861

**GLU (mmol/L)**	5.33 ± 0.934	9.01 ± 15.51	6.11 ± 1.24	0.341
**LVEF**	0.70 ± 0.07	0.67 ± 0.10	0.71 ± 0.06	0.269
**TG**	1.38 ± 0.73	1.77 ± 1.29	2.04 ± 1.36	0.125
**HDL-C**	1.29 ± 0.20	1.28 ± 0.30	1.23 ± 0.33	0.737
**LDL-C**	2.44 ± 0.64	2.52 ± 0.73	2.7368 ± 0.88	0.391

For continuous data conformed to normality, data was described as mean ± standard deviation (mean ± SD). *P < 0.05; **P < 0.01; ***P < 0.001. Body mass index (BMI), Systolic blood pressure (SBP), White blood cells (WBC), Hemoglobin (Hb), Alamine aminotransferase (ALT), Aspartate aminotransferase (AST), Blood urea nitrogen (BUN), Uric acid (UA), Glucose (GLU), Left ventricular ejected fraction (LVEF), triglyceride (TG), High-density lipoprotein cholesterol (HDL-C), Low-density lipoprotein cholesterol (LDL-C).
